# Neuron‐Derived MIF Engages VCAM1 to Fuel a Self‐Amplifying CXCL8 Loop That Drives Perineural Invasion and Metastasis in Gastric Cancer

**DOI:** 10.1002/advs.76195

**Published:** 2026-06-22

**Authors:** Xunjun Li, Zhongya Zhai, Haiyi Yu, Renjie Qiu, Fengyu Li, Luxi Xiao, Boxi Huang, Huiying Liu, Jiayong He, Bowen Cai, Jiawen Chen, Jiang Yu, Guoxin Li, Tao Chen

**Affiliations:** ^1^ Department of General Surgery & Guangdong Provincial Key Laboratory of Precision Medicine for Gastrointestinal Tumor Nanfang Hospital Southern Medical University Guangzhou Guangdong Province China; ^2^ Department of Thyroid And Breast Surgery Shenzhen Hospital Southern Medical University Shenzhen Guangdong Province China; ^3^ Cancer Center of Beijing Tsinghua Changgung Hospital School of Clinical Medicine Tsinghua Medicine Tsinghua University Beijing China; ^4^ Department of Gastrointestinal and Hernia Surgery Ganzhou Hospital‐Nanfang Hospital Southern Medical University Ganzhou Jiangxi Province China

**Keywords:** gastric cancer, neural‐tumor interaction, perineural invasion, tumor proliferation and invasion

## Abstract

**Background**: Perineural invasion (PNI) is common in gastric cancer and predicts poor prognosis, but the molecular mechanisms underlying tumour–nerve crosstalk remain unclear.

**Methods**: Transcriptomic data from TCGA and GEO were integrated to identify PNI regulators, with validation in surgical specimens. Mechanistic studies used tumor–neuron Transwell co‐culture, siRNA/lentiviral perturbation, Western blot, immunofluorescence, ELISA, co‐immunoprecipitation, and GST pull‐down. Functional effects were assessed via CCK‐8/Transwell assays, a sciatic nerve invasion xenograft model, and an orthotopic gastric cancer model with neuron‐specific MIF conditional knockout mice.

**Results**: High CXCL8/VCAM1 expression correlates with poor overall survival, distant metastasis, and is upregulated in PNI‐positive tumours. Mechanistically, CXCL8 promotes neuronal MIF expression, while MIF directly binds VCAM1 and enhances tumour CXCL8 expression. VCAM1‐mediated CXCL8 upregulation drives tumour proliferation and invasion. In vivo, blocking CXCL8/VCAM1 or neuron‐specific MIF deletion reduces tumour growth and neural invasion.

**Conclusions**: A MIF–VCAM1–CXCL8 positive‐feedback axis linking neural activity to gastric cancer progression and PNI is identified, providing prognostic biomarkers and actionable therapeutic targets.

## Graphical abstract

Neuron‐derived MIF binds VCAM1 on gastric cancer cells and activates ERK/STAT3 signaling, leading to CXCL8 transcription and secretion. Tumor‐derived CXCL8 subsequently stimulates neuronal CXCR2 to enhance MIF production, establishing a self‐amplifying MIF–VCAM1–CXCL8 positive‐feedback loop that promotes perineural invasion, tumor progression, and metastasis in gastric cancer.

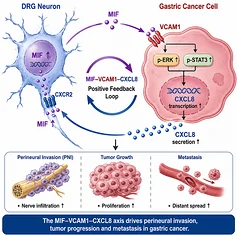

## 1 Introduction

Gastric cancer remains a leading cause of global cancer‐related mortality, accounting for ∼7.7% of all cancer deaths [1], with perineural invasion (PNI) emerging as a critical pathological feature that worsens patient outcomes [2, 3, 4]. Defined as tumor cell infiltration into nerve fibers and perineurium [5, 6], PNI occurs in up to 75.6% of gastric cancer cases [7] and serves as an independent prognostic factor—linking to advanced tumor stage, increased local recurrence, and a 1.55‐fold higher mortality risk even after adjusting for confounding variables [8]. Despite its clinical significance, the molecular mechanisms governing the bidirectional crosstalk between tumor cells and peripheral nerves during PNI remain largely elusive, representing a major barrier to developing targeted therapies for high‐risk patients.

PNI is no longer regarded as a passive anatomical coincidence but rather an active, signal‐driven interplay [9]. In other malignancies, such as prostate and pancreatic cancer, axon guidance molecules and chemokines have been established as pivotal mediators of tumor‐nerve crosstalk [10, 11, 12]. Tumor cells secrete neurotrophic factors [13, 14]) to recruit and rewire nerves, whereas neural components secrete neurotransmitters [15, 16] and cytokines [17] that augment tumor aggressiveness. Current studies on PNI are predominantly concentrated in pancreatic, and prostate cancers, whereas research on gastric cancer PNI is still in its infancy [2, 3, 4, 8]. Existing reports have only identified a few independent molecules (such as NGF, 5‐HT) involved in gastric cancer‐nerve interaction [13, 14], but the synergistic regulatory network and self‐amplifying signaling pathways mediated by multiple molecules remain completely uncharacterized.

Chemokines and adhesion molecules are central to tumor‐stromal interactions, including tumor‐nerve crosstalk [18, 19]. CXCL8(interleukin‐8) is a key pro‐inflammatory chemokine that is highly expressed in gastric cancer, and existing studies confirm that CXCL8 promotes gastric cancer metastasis and angiogenesis through PI3K/AKT and MAPK pathways [20, 21], but its function in activating peripheral neurons and initiating PNI has never been investigated. Vascular cell adhesion molecule 1(VCAM1) is a classic adhesion molecule that mediates tumor‐endothelial cell adhesion and immune cell infiltration in breast and pancreatic cancers [22, 23], and a recent study revealed that VCAM1 promotes gastric cancer progression via the AKT‐mTOR‐CXCL1 axis [24], but its role as a receptor for neural cytokines and its interaction with CXCL8 in PNI remain unclear. Additionally, macrophage migration inhibitory factor(MIF), a pleiotropic cytokine involved in inflammation and tumorigenesis [25, 26], regulates cell adhesion and proliferation in multiple cancers, but its role in gastric cancer PNI and tumor‐nerve communication has not been reported. Most importantly, no existing studies have explored the synergistic crosstalk and self‐amplifying feedback loop formed by CXCL8, VCAM1, and MIF in gastric cancer PNI, which is the core scientific question to be solved in this study.

To address these critical knowledge gaps, we hypothesized that a coordinated signaling axis involving CXCL8, VCAM1, and MIF orchestrates bidirectional tumor‐nerve crosstalk to promote PNI in gastric cancer. Given that research on the crosstalk between the nervous system and gastric cancer remains very limited, especially in the setting of tumor perineural invasion. Furthermore, most studies have focused primarily on classical molecules such as neurotransmitters. In this study, we integrated transcriptomic profiling, in vitro tumor‐neuron co‐culture models, and in vivo xenograft/neuron‐specific conditional knockout systems to systematically dissect the composition and functional relevance of this putative axis. Our results uncover a novel MIF‐VCAM1‐CXCL8 positive‐feedback loop that couples neural activity to tumor proliferation, metastasis, and perineural invasion. This work not only delineates the molecular underpinnings of PNI in gastric cancer but also identifies prognostic biomarkers and actionable therapeutic targets, offering new opportunities to improve clinical outcomes for patients with PNI‐positive gastric cancer.

## 2 Materials and Methods

### 2.1 Datasets

Raw transcriptomic profiles and corresponding clinicopathological annotations of TCGA‐STAD and GSE62254 were downloaded from the Genomic Data Commons (GDC) portal and Gene Expression Omnibus (GEO), respectively. Only samples with available overall survival information and pathological PNI annotation were included in the integrated public‐cohort analyses. For cross‐cohort analyses, gene expression matrices were harmonized at the gene‐symbol level and subsequently corrected for batch effects before downstream modeling. The institutional retrospective cohort included surgically resected gastric adenocarcinoma patients collected at Nanfang Hospital, Southern Medical University between October 2004 and September 2011. Inclusion criteria for the retrospective cohort were: confirmed primary gastric adenocarcinoma by histopathology; underwent curative gastrectomy with D2 lymphadenectomy; available complete clinicopathological data; available pathological report with clear documentation of PNI status; minimum follow‐up duration of at least 100 days after surgery. Exclusion criteria were: patients who received neoadjuvant chemotherapy or radiotherapy before surgery; patients with synchronous or metachronous malignancies; patients lost to follow‐up within 100 days after surgery. A total of 356 patients were initially screened, and 12 patients were excluded due to early loss to follow‐up within 100 days. The final analysis included 344 patients, comprising 100 patients with PNI and 244 patients without PNI. All patients who died during follow‐up, including those who died within 100 days postoperatively, were included with their actual survival time recorded. Finally, TCGA and GEO cohorts were combined as a combined cohort (n = 362), and the Nanfang cohort comprised 344 patients. All analyses of public data followed the data‐use policies of the respective repositories.

### 2.2 Clinical Samples and Ethics

Fresh surgical specimens (tumor and paired adjacent normal tissue) were obtained from patients undergoing gastrectomy at Nanfang Hospital, Southern Medical University. A total of 60 gastric cancer patients with documented PNI status were enrolled in this part of analysis. Among them, 32 patients with paired fresh tumor and adjacent normal tissues were used for qRT‐PCR analysis, and all 60 patients with formalin‐fixed paraffin‐embedded (FFPE) tissue sections were subjected to immunohistochemical (IHC) staining. Sample collection and use were approved by the Institutional Review Board of Nanfang Hospital (Ethics approval no. FM202410270009) and written informed consent was obtained from all patients prior to inclusion.

### 2.3 Cell Culture and Authentication

Human gastric cancer cell lines(MGC‐803, MKN‐45, SGC‐7901, AGS, NCC‐24, SNU‐216, HGC‐27) and normal gastric epithelial cell line GES‐1 were obtained from the Cell Bank of the Chinese Academy of Sciences(Shanghai, China). All cell lines were authenticated by short tandem repeat(STR) profiling(Genetic Testing Biotechnology Co., Ltd., Suzhou, China) and tested negative for mycoplasma contamination(Mycoplasma Detection Kit, Beyotime, Shanghai, China). Of note, NCC‐24 is a diffuse‐type gastric cancer cell line established from a primary signet‐ring cell carcinoma. SNU‐216 was established from a lymph node metastasis of moderately differentiated tubular adenocarcinoma; although its Lauren classification was not explicitly designated in the original report, it displays highly invasive properties consistent with aggressive gastric cancer subtypes.

Cells were cultured in RPMI 1640 medium(Gibco, USA) supplemented with 10% fetal bovine serum(FBS, Gibco, USA) and 1% penicillin‐streptomycin(Gibco, USA) at 37°C in a humidified atmosphere with 5% CO_2_. Medium was refreshed every 2–3 days, and cells were passaged when confluency reached 80%–90% using 0.25% trypsin‐EDTA(Gibco, USA).

### 2.4 siRNA Transfection and Lentiviral Infection

siRNA of CXCL8, VCAM1 and MIF were purchased from GeneChem (Shanghai,China). Cells were seeded in 6‐well plates(2×10^5^ cells/well) 24 h prior to transfection. Transfection was performed using Lipofectamine 3000(Invitrogen, USA) according to the manufacturer's protocol, with a final siRNA concentration of 50 nM. Cells were harvested 48–72 h post‐transfection for downstream assays. VCAM1 overexpression(OE) and short hairpin RNA(shVCAM1) lentiviruses were constructed by GeneChem(Shanghai, China). Cells were seeded in 6‐well plates(1×10^5^ cells/well) and infected with lentivirus at a multiplicity of infection(MOI) of 10, supplemented with 5 µg/mL polybrene(Sigma‐Aldrich, USA). Stable cell lines were selected with 2 µg/mL puromycin(Sigma‐Aldrich, USA) for 2 weeks, and VCAM1 expression was verified by Western blot.

### 2.5 Immunohistochemistry (IHC)

A total of 60 gastric cancer samples were included in the IHC analysis. Formalin‐fixed paraffin‐embedded(FFPE) tissue blocks were sectioned at 4 µm. Sections were deparaffinized, rehydrated, and subjected to antigen retrieval in citrate buffer(pH 6.0) or EDTA buffer(pH 9.0) as appropriate for each antibody. Endogenous peroxidase activity was blocked with 3% hydrogen peroxide. Sections were incubated with primary antibodies overnight at 4°C. Primary antibodies used include: Anti‐MIF, Anti‐CXCL8, Anti‐VCAM1, Anti‐CXCR2, Anti‐S100. Sections were incubated with appropriate HRP‐conjugated secondary antibodies, developed with DAB, counterstained with hematoxylin, dehydrated and mounted. Staining intensity and positive cell percentages were scored independently by two pathologists blinded to clinical outcomes; discrepancies were resolved by joint review.

### 2.6 Co‐Immunoprecipitation(Co‐IP) Assay

Cells were lysed in IP lysis buffer(Beyotime, Shanghai, China) supplemented with protease and phosphatase inhibitors(Roche, Switzerland) on ice for 30 min. Lysates were centrifuged at 12,000×g for 15 min at 4°C to collect supernatants. 500 µg of total protein was incubated with 2 µg of anti‐VCAM1(Abcam, USA) or anti‐MIF(Abcam, USA) antibody overnight at 4°C, followed by addition of 20 µL Protein A/G Plus Agarose Beads(Santa Cruz Biotechnology, USA) and incubation for 4 h at 4°C with gentle rotation. Beads were washed 5 times with cold IP wash buffer (Beyotime, Shanghai, China), resuspended in 2×SDS loading buffer, and boiled at 95°C for 10 min to elute immunoprecipitated proteins. Samples were analyzed by Western blot.

### 2.7 Western Blotting

Cell or tissue lysates were prepared in lysis buffer supplemented with protease and phosphatase inhibitors. Protein concentrations were determined using a BCA assay kit. Equal amounts of protein were separated by SDS‐PAGE, transferred to PVDF membranes, blocked with 5% non‐fat milk, and probed with primary antibodies, including Anti‐GAPDH, Anti‐CXCL8, Anti‐CXCR2, Anti‐VCAM1, Anti‐CXCR1, Anti‐FLAG, Anti‐His, Anti‐ERK1/2, Anti‐phospho‐ERK1/2, Anti‐STAT3, and Anti‐phospho‐STAT3. HRP‐conjugated secondary antibodies were used for detection, and chemiluminescent signals were captured using a digital imaging system. Quantification was performed using ImageJ software and normalized to loading controls.

### 2.8 GST Pull‐Down Assay

GST‐VCAM1(full‐length or truncated domains) and His‐MIF plasmids were transformed into E. coli BL21(DE3) cells(TransGen Biotech, Beijing, China). Protein expression was induced with 0.5 mM isopropyl β‐D‐1‐thiogalactopyranoside(IPTG) at 37°C for 4 h. GST‐tagged proteins were purified using Glutathione Sepharose 4B Beads(Boxin Biotech, China), and His‐tagged proteins were purified using Ni‐NTA Agarose Beads(Boxin Biotech, China) according to manufacturers’ protocols. 20 µg of GST‐VCAM1(or GST alone as control) was incubated with 10 µg of His‐MIF in binding buffer(20 mM Tris‐HCl pH 7.5, 150 mM NaCl, 1 mM EDTA, 0.5% Triton X‐100) at 4°C for 4 h. Glutathione Sepharose 4B Beads were added and incubated for another 2 h, followed by 5 washes with binding buffer. Bound proteins were eluted with 2×SDS loading buffer and analyzed by Western blot with anti‐His(Proteintech, China) and anti‐GST(Abcam, USA) antibodies.

### 2.9 Enzyme‐Linked Immunosorbent Assay (ELISA)

Culture supernatants of DRG neurons were collected and centrifuged at 10,000×g for 10 min to remove debris. MIF ELISA Kit(R&D Systems, USA) was used according to the manufacturer's instructions. Briefly, 100 µL of standard or sample was added to pre‐coated 96‐well plates and incubated at room temperature for 2 h. Plates were washed 4 times with wash buffer, followed by addition of 100 µL of detection antibody and incubation for 1 h. After washing, 100 µL of streptavidin‐HRP was added and incubated for 30 min. Plates were washed again, and 100 µL of substrate solution was added for 20 min in the dark. The reaction was stopped with stop solution, and absorbance was measured at 450 nm using a microplate reader (Thermo Fisher Scientific, USA).

### 2.10 Tumor–Neuron Transwell Co‐Culture System

Sprague‐Dawley rats(1–3 days old) were euthanized, and dorsal root ganglia (DRG) were dissected under sterile conditions. DRG neurons were isolated by digestion with 0.25% trypsin‐EDTA for 15 min at 37°C, triturated to single‐cell suspension, and seeded in poly‐L‐lysine(Sigma‐Aldrich, USA)‐coated Transwell inserts(pore size 0.4 µm, Corning, USA) at 5×10^4^ cells/insert in Neurobasal Medium (Gibco, USA) supplemented with 2% B27(Gibco, USA) and 50 ng/mL nerve growth factor(NGF, R&D Systems, USA). For “tumor‐to‐neuron” co‐culture (Figure 4B), tumor cells(SNU‐216 or NCC‐24) were seeded in the upper chamber(2×10^5^ cells/well) of 6‐well Transwell plates, and DRG neurons(seeded in inserts) were placed in the lower chamber. For “neuron‐to‐tumor” co‐culture (Figure 5A), DRG neurons were seeded in the upper chamber(inserts), and tumor cells were seeded in the lower chamber (2×10^5^ cells/well). Co‐culture medium was RPMI 1640:Neurobasal Medium(1:1) supplemented with 5% FBS and 1% B27. Cells were co‐cultured for 24–72 h, and samples were collected for Western blot, qRT‐PCR, or ELISA.

The purity of primary DRG neuronal cultures was validated by immunofluorescence staining using the neuron‐specific marker Tuj1 (anti‐Tuj1, 1:500, Abcam, USA). For co‐localization analysis, DRG neurons were fixed, permeabilized, and incubated with primary antibodies against MIF (1:200, Abcam, USA) and Tuj1 (1:500, Abcam, USA), followed by Alexa Fluor 488‐conjugated anti‐rabbit IgG and Alexa Fluor 594‐conjugated anti‐mouse IgG secondary antibodies. Nuclei were counterstained with DAPI. Images were captured using a laser scanning confocal microscope (Zeiss LSM 980).

For CXCR2 antagonist pretreatment experiments, DRG neurons were seeded in the upper Transwell inserts and cultured for 24 h. Neurons were then pretreated with 10 µM SB225002(an inhibitor of CXCL8 and CXCR2 binding; MCE, HY‐16711) or equal volume of DMSO for 6 h at 37°C. After pretreatment, unbound drug was removed by washing three times with pre‐warmed PBS. Pretreated DRG neurons were co‐cultured with SNU‐216 cells in the Transwell system for 72 h. SNU‐216 cells in the lower chamber were harvested for Western blot analysis of VCAM1 and CXCL8 expression.

### 2.11 CCK‐8 Proliferation Assay

Cells were seeded in 96‐well plates at 2×10^3^ cells/well and cultured for 0, 24, 48, or 72 h. For rescue experiments, cells were transfected with siRNA or lentivirus 24 h prior to seeding. At each time point, 10 µL of CCK‐8 reagent(Dojindo, Japan) was added to each well, and plates were incubated at 37°C for 2 h. Absorbance at 450 nm was measured using a microplate reader(Thermo Fisher Scientific, USA). Each group had 6 replicate wells, and experiments were repeated 3 times independently.

### 2.12 Transwell Migration and Invasion Assays

Cells(5×10^4^ cells/well) were resuspended in serum‐free medium and seeded in the upper chamber of Transwell inserts(pore size 8 µm, Corning, USA). The lower chamber was filled with 600 µL of medium containing 10% FBS. For co‐culture experiments, DRG neurons were seeded in the lower chamber 24 h prior to tumor cell seeding. After 24 h of incubation, non‐migrated cells on the upper surface were removed with a cotton swab, and migrated cells on the lower surface were fixed with 4% paraformaldehyde, stained with 0.1% crystal violet, and counted under a microscope(5 random fields/insert, Olympus, Japan).

Transwell inserts were pre‐coated with 50 µL of Matrigel(1:8 dilution in serum‐free medium, Corning, USA) and incubated at 37°C for 4 h to form a gel. Cells (1×10^5^ cells/well) were seeded in the upper chamber, and the rest of the procedure was the same as the migration assay. Incubation time was extended to 48 h for invasion assays.

### 2.13 Rescue Experiment

For rescue experiments, gastric cancer cells were transfected with siRNA targeting VCAM1 or negative control siRNA. After 24 h, cells were treated with recombinant MIF protein (50 ng/mL) for 72 h. The protein levels of p‐ERK, ERK, p‐STAT3, STAT3, and CXCL8 were detected by Western blot.

### 2.14 Animal Experiments

All animal studies were approved by the Ethics Committee of Nanfang Hospital, Southern Medical University(IACUC‐LAC‐20230224‐005) and were conducted following institutional guidelines for animal care.

### 2.15 Sciatic Nerve Invasion Model

Six‐week‐old BALB/c nude mice were acclimated for one week before use. The sciatic nerve PNI model was established as previously described and adapted from Deborde et al. [27]. SNU‐216 GC cells were resuspended at 1 × 10^6 cells/50 µL and mixed 1:1 with Matrigel on ice. Mice were anesthetized (isoflurane), and 50 µL of cell suspension was injected adjacent to the exposed sciatic nerve. Animals were monitored daily and euthanized at humane endpoints or 4 weeks post‐implantation. Tumor and sciatic nerve tissues were harvested for histology and molecular assays.

PNI was evaluated according to the gold‐standard pathological definition established by the field [6], which defines PNI as tumor cell infiltration into any of the three layers of the nerve sheath (epineurium, perineurium, or endoneurium). This definition is completely consistent with the diagnostic criteria for PNI in our human gastric cancer clinical cohort. All sciatic nerve tissues were serially sectioned and stained with the pan‐neuronal marker S100, to accurately delineate the complete boundary of the nerve fascicle and the tumor‐invaded region.

Given that tumor cells were inoculated directly adjacent to the sciatic nerve sheath, all mice developed histologically identifiable tumor‐nerve interaction; thus, PNI incidence (presence/absence) was not used as an endpoint. Instead, we focused on the severity and extent of nerve invasion, and performed all PNI assessments in a fully blinded, standardized manner:We quantified PNI severity using a dual standardized metric system: ① Quantitative indicators: The percentage of the area where tumor cells invade the neural tissue relative to the total cross‐sectional area of the nerve bundle(measured by ImageJ); ② Semi‐quantitative severity scoring (0–3 scale, corresponding to the degree of nerve parenchyma destruction): 0 = no tumor invasion into any nerve sheath layer; 1 = tumor only attached to the epineurium without invasion; 2 = tumor infiltrated into the perineurium; 3 = tumor invaded the endoneurium and destroyed normal nerve structure.

### 2.16 Generation of Neuron‐Specific Mif Knockout Mice

To achieve neuron‐specific deletion of Mif, we employed the Cre‐loxP recombination system. Two transgenic strains were obtained from Shanghai Model Organisms Center(SMOC): C57BL/6Smoc‐Miftm1(flox)Smoc (floxed Mif) and C57BL/6Smoc‐Nesem1(IRES‐CreERT2) Smoc(Nestin‐CreERT2). Nes‐CreERT2 mice were crossed with Mif^fl/fl^ mice to generate offspring of two genotypes: C57BL/6‐Nes‐Cre^+/−^; Mif^fl/fl^ (experimental group) and C57BL/6‐Nes‐Cre^−/−^; Mif^fl/fl^ (control group) Genomic DNA was extracted from tail biopsies, and genotyping was performed by PCR. To verify the efficiency and specificity of Mif knockout in neurons, DRG were isolated from neuron‐specific Mif knockout (cKO) and wild‐type (WT) mice. The mRNA and protein levels of MIF in DRG tissues were detected by quantitative real‐time PCR (qPCR) and Western blot analysis, respectively.

The Sleeping Beauty(SB) transposon system was employed to induce spontaneous gastric cancer in neuron‐specific Mif conditional knockout mice (Nes‐Cre^+^; Mif^fl/fl^) and control mice(Nes‐Cre^−^; Mif^fl/fl^), relying on co‐expression of two oncogenes, AKT and MYC, which synergistically drive gastric epithelial cell transformation. The transposon vector(pT3‐CAG‐AKT‐IRES‐MYC, purchased from ShouZheng Co., Ltd., Wuhan, China) and transposase plasmid(pCMV‐SB100X) were mixed in a molar ratio of 10:1 in sterile PBS, with a final total plasmid concentration of 2 µg/µL. A volume of 50 µL of the plasmid mixture was injected into the gastric submucosa of each mouse using a 30‐gauge microsyringe(Hamilton, USA) under stereomicroscopic guidance. The injection site was localized to the anterior wall of the gastric body to ensure targeted oncogene delivery to gastric epithelial stem cells.

To validate the neuron‐specificity of Cre recombinase activity, B6.Cg‐Gt(ROSA)26Sor^tm9(CAG‐tdTomato)Hze^/J reporter mice (Jackson Laboratory, USA) were crossed with Nestin‐CreERT2 mice to generate double‐transgenic offspring (Nes‐Cre^+^;tdTomato^+^). At 6 weeks of age, double‐transgenic mice received intraperitoneal injections of tamoxifen (75 mg/kg body weight) once daily for 5 consecutive days to induce Cre recombinase expression. Two weeks after the last injection, mice were euthanized, and gastric tissues were harvested for frozen section preparation and immunofluorescence analysis.

### 2.17 Bioinformatics Analysis

First, transcriptomic data(TCGA‐STAD RNA‐seq and GSE62254 microarray) and clinical information were downloaded, preprocessed(background correction, normalization, probe annotation), and filtered to retain high‐confidence expressed genes. Next, differentially expressed genes(DEGs) were screened between PNI‐positive and PNI‐negative groups, as well as between “CXCL8 high + VCAM1 high” and “CXCL8 low + VCAM1 low” groups.

To prioritize key genes, PNI‐related candidate genes were collected from Pathway Studio, and a protein‐protein interaction(PPI) network was constructed via STRING. Hub genes were identified using Cytoscape's CytoHubba plugin, and core candidates(CXCL8, VCAM1, etc.) were determined by intersecting hub genes with PNI‐related DEGs.

Functional enrichment analyses (GO and KEGG) were performed to explore biological processes and pathways associated with the core gene signature. Weighted Gene Co‐expression Network Analysis(WGCNA) was applied to construct co‐expression modules and correlate them with clinical traits(e.g., overall survival, PNI status) to extract trait‐relevant core genes.

To explore the association of the MIF‐VCAM1‐CXCL8 axis with the tumor microenvironment, the cell composition of the gastric cancer TME was estimated using the CIBERSORTx algorithm based on the transcriptomic data of the TCGA‐STAD cohort. Briefly, gene expression profiles were deconvolved to obtain the relative proportions of 12 major cell types, including B cells, CD4^+^ T cells, CD8^+^ T cells, NK cells, macrophages, inflammatory monocytes, mast cells, plasma cells, endothelial cells, cancer‐associated fibroblasts (CAFs), pericytes/VSMCs, and natural killer cells. For each of the three core genes (CXCL8, VCAM1, and MIF), patients were stratified into high‐ and low‐expression groups using the optimal cut‐off value determined by maximizing the area under the receiver operating characteristic curve (AUC) for overall survival prediction. The differences in the proportions of each cell type between the high and low groups were compared using the Wilcoxon rank‐sum test. All analyses were performed in R(version 4.0.5) with relevant packages including ‘CIBERSORTx’, ‘ggplot2’, and ‘dplyr’.

Finally, survival analyses were performed using the ‘survival’ and ‘survminer’ packages. Kaplan‐Meier curves were generated and compared using the log‐rank test. The optimal cutoff values for gene expression stratification were determined by maximizing the area under the receiver operating characteristic (ROC) curve for overall survival prediction.

The detailed of bioinformatics analysis process can be found in the “Methods” section of the supplementary file.

### 2.18 Statistical Analysis

All in vitro experiments were repeated at least three times independently, and data are presented as mean ± standard deviation (SD). Statistical analyses were performed using GraphPad Prism v8 and R software (version 4.0.5). A two‐sided P < 0.05 was considered statistically significant.

Comparisons between two independent groups: normally distributed data were analyzed using the two‐tailed Student's t‐test; non‐normally distributed data were analyzed using the Wilcoxon rank‐sum test. This method was applied to Western blot quantification, ELISA, CCK‐8, Transwell migration/invasion, and colony formation assays.

Comparisons among multiple groups: one‐way analysis of variance (ANOVA) followed by Tukey's post hoc test was used for normally distributed data with equal variances. This method was applied to dose‐response experiments and multi‐group intervention studies.

Paired comparisons: paired comparisons between tumor and matched adjacent normal tissues were performed using the paired Wilcoxon signed‐rank test. This method was applied to qRT‐PCR and IHC analyses of paired clinical samples.

Categorical data analysis: associations between gene expression and clinicopathological characteristics were analyzed using the chi‐square test or Fisher's exact test (when expected frequencies < 5).

Survival analysis: Kaplan‐Meier curves were generated and survival differences were assessed using the log‐rank test. Univariate and multivariate Cox proportional hazards regression models were used to identify independent prognostic factors. The optimal cutoff values for gene expression stratification were determined by maximizing the area under the receiver operating characteristic (ROC) curve.

Detailed antibody information, primer sequences, software, and instruments are provided in Table S1.

## 3 Results

### 3.1 Perineural Invasion is Associated With Adverse Prognosis in Patients With Gastric Cancer

**FIGURE 1:**
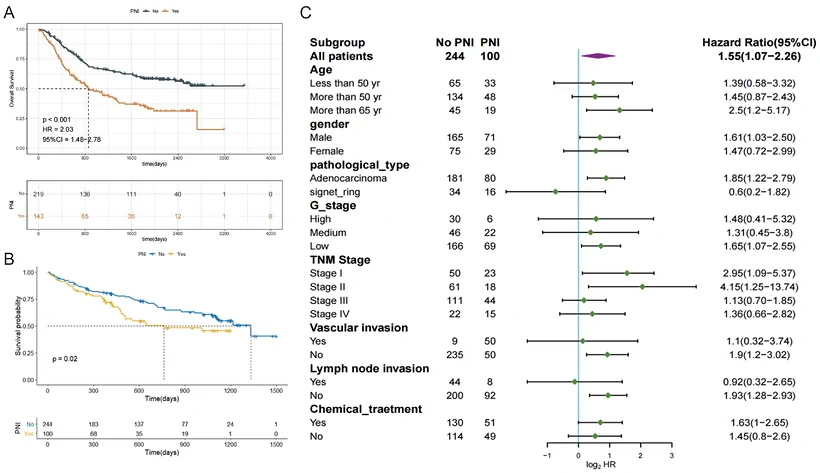
Perineural invasion is associated with adverse prognosis in patients with gastric cancer. (A) Kaplan–Meier overall survival (OS) curves for PNI‐positive (PNI+) versus PNI‐negative (PNI–) patients in the combined gastric cancer cohort (TCGA‐STAD + GSE62254). Numbers at risk are shown beneath the plot. Survival differences were assessed by the log‐rank test. (B) Kaplan–Meier OS curves for PNI+ versus PNI– patients in the Nanfang Hospital gastric cancer cohort. Numbers at risk are shown; comparison by log‐rank test. (C) Forest plot showing risk estimates (hazard ratios and 95% confidence intervals) for PNI+ versus PNI– across clinical subgroups in Nanfang Hospital cohort. Risk estimates were derived from stratified and multivariable Cox proportional hazards models as indicated. Statistical significance is denoted by *P < 0.05.

Kaplan‐Meier analysis demonstrated that PNI‐positive patients had significantly worse overall survival than PNI‐negative patients in the combined cohort (log‐rank P < 0.001; Figure 1A) and in the Nanfang cohort (log‐rank P = 0.02; Figure 1B). The above results confirm that patients with positive PNI have a poorer prognosis.

To evaluate the independent prognostic effect of PNI, we performed multivariable Cox proportional hazards regression adjusting for age, sex, histologic subtype, tumor stage, differentiation, vascular invasion, lymph node status and receipt of adjuvant chemotherapy. After adjustment, PNI remained an adverse prognostic factor. Subgroup Cox analyses stratified by age, sex, histologic type, differentiation, TNM stage, vascular invasion, nodal status and chemotherapy exposure showed that PNI was associated with increased mortality risk in the majority of clinical strata (Figure 1C).

### 3.2 Network‐ and Expression‐Based Analyses Identify PNI‐Associated Hub Genes

**FIGURE 2:**
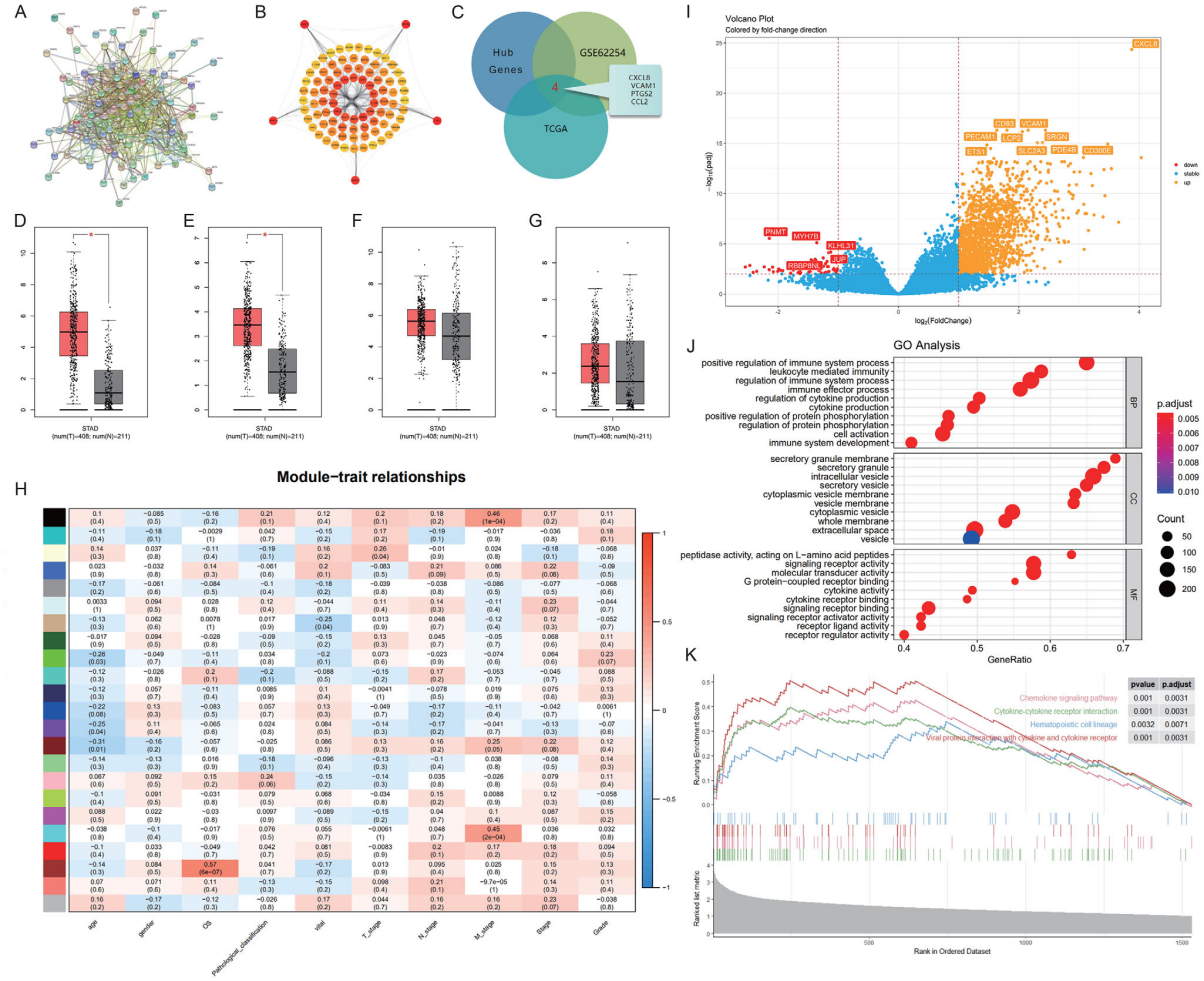
Network‐ and expression‐based analyses identify PNI‐associated hub genes. (A) STRING protein–protein interaction network constructed from genes associated with neural invasion/neuro‐aggression. Edge thickness represents interaction confidence. (B) Cytoscape analysis of the STRING network highlighting hub genes. Hub genes are color‐coded and labeled. (C) Venn diagram showing the intersection among differentially expressed genes (PNI‐positive vs. PNI‐negative) from TCGA and GSE62254, and the Cytoscape‐identified hub gene set. (D–G) Differential RNA expression of CXCL8, VCAM1, CCL2 and PTGS2 in tumor versus normal tissue (boxplots). Statistical comparisons were performed using the Wilcoxon rank‐sum test; *P < 0.05. (H) Weighted gene co‐expression network analysis (WGCNA) performed on the differential gene set; module–trait relationships with clinical variables (e.g., stage, PNI) are displayed (correlation coefficients and P values shown). (I) Volcano plot of differential expression comparing tumors with high versus low CXCL8/VCAM1 expression (genes meeting fold‐change and adjusted P value thresholds are highlighted). (J) Top Gene Ontology (GO) biological processes enriched among the differential gene set (displayed with enrichment score and corrected P values). (K) Top KEGG pathways significantly enriched among the differential gene set (pathway names, enrichment ratios and FDR‐adjusted P values shown).

To further explore the potential regulatory factors that cause PNI and its associated adverse prognosis, we extracted gene annotations related to “perineural invasion” and “neuron–tumour interactions” from Pathway Studio. The resulting gene set was submitted to STRING to construct a protein‐protein interaction (PPI) network (Figure 2A). Using network topology metrics (including degree and MCC) and Cytoscape‐based analyses, we defined a set of network‐central hub genes (Figure 2B) that served as a PNI‐related candidate panel for subsequent filtering and validation.

To prioritise gastric cancer‐specific mediators among the Pathway Studio PNI candidates, we performed PNI‐positive tumors versus PNI‐negative tumors differential expression analyses separately in TCGA–STAD cohort and GSE62254 cohort using |log2FC| > 1 and Benjamini‐Hochberg adjusted P < 0.05. The PNI‐specific DEG sets were intersected with the PNI hub‐gene list (Venn analysis): CXCL8, VCAM1, PTGS2 and CCL2 (Figure 2C). We compared these genes between tumor tissues and normal tissues in the TCGA‐STAD dataset. Only CXCL8 and VCAM1 showed statistically significant differences (Figure 2D‐G). These findings suggest that the upregulation of CXCL8 and VCAM1 may be key genes mediating the occurrence of PNI in GC and the progression of the tumor. Therefore, we conducted our research focusing on these two genes.

In the TCGA‐STAD cohort, based on the optimal cut‐off value of each gene, we divided the patients into two groups: those with simultaneous high expression of CXCL8 and VCAM1, and those with simultaneous low expression of both. We then conducted a differential gene analysis on these groups. Based on these differential genes, we performed GO, KEGG, and WGCNA analyses. Differential expression analysis revealed that the double‐high group exhibited significant upregulation of CXCL8, VCAM1, CD93, SRGN, PECAM1, LCP2, ETS1, SLC2A3, PDE4B and CD300E, whereas PNMT, MYH7B, KLHL31, JUP and RBBP8NL were among the significantly downregulated genes (Figure 2I). Functional enrichment analysis of this gene set showed significant enrichment (adjusted P < 0.01) for GO terms related to immune activation, including positive regulation of immune system process (p.adjust = 0.006), leukocyte‐mediated immunity (p.adjust = 0.008), regulation of cytokine production (p.adjust = 0.009) and cytokine activity (p.adjust = 0.010) (Figure 2J). At the molecular function and cellular component levels, the genes were enriched for signalling receptor activity, cytokine receptor binding, secretory granule membrane and extracellular space, suggesting predominant involvement in secretory and receptor‐mediated signalling processes. KEGG analysis further identified “cytokine‐cytokine receptor interaction” as a significantly enriched pathway (p.adjust = 0.0031), implicating CXCL8, VCAM1 and their receptors as central mediators of neuron–tumour crosstalk (Figure 2K).

To assess the clinical relevance of these differentially expressed genes, we applied WGCNA and correlated the resulting modules with clinicopathological features (age, sex, overall survival, histological subtype, TNM stage and differentiation grade). These different genes showed a significant positive correlation with overall survival(OS) and distant metastasis (M stage) (Figure 2H). This is consistent with the survival analysis results shown in Figure 1A,B. Collectively, these integrative analyses establish CXCL8 and VCAM1 as key candidate mediators in the context of gastric cancer PNI and support their potential role in orchestrating immune/cytokine signalling and promoting tumour invasion and dissemination.

### 3.3 CXCL8 and VCAM1 are Upregulated in Gastric Cancer Tissues and Correlate With Poor Clinical Outcome

**FIGURE 3:**
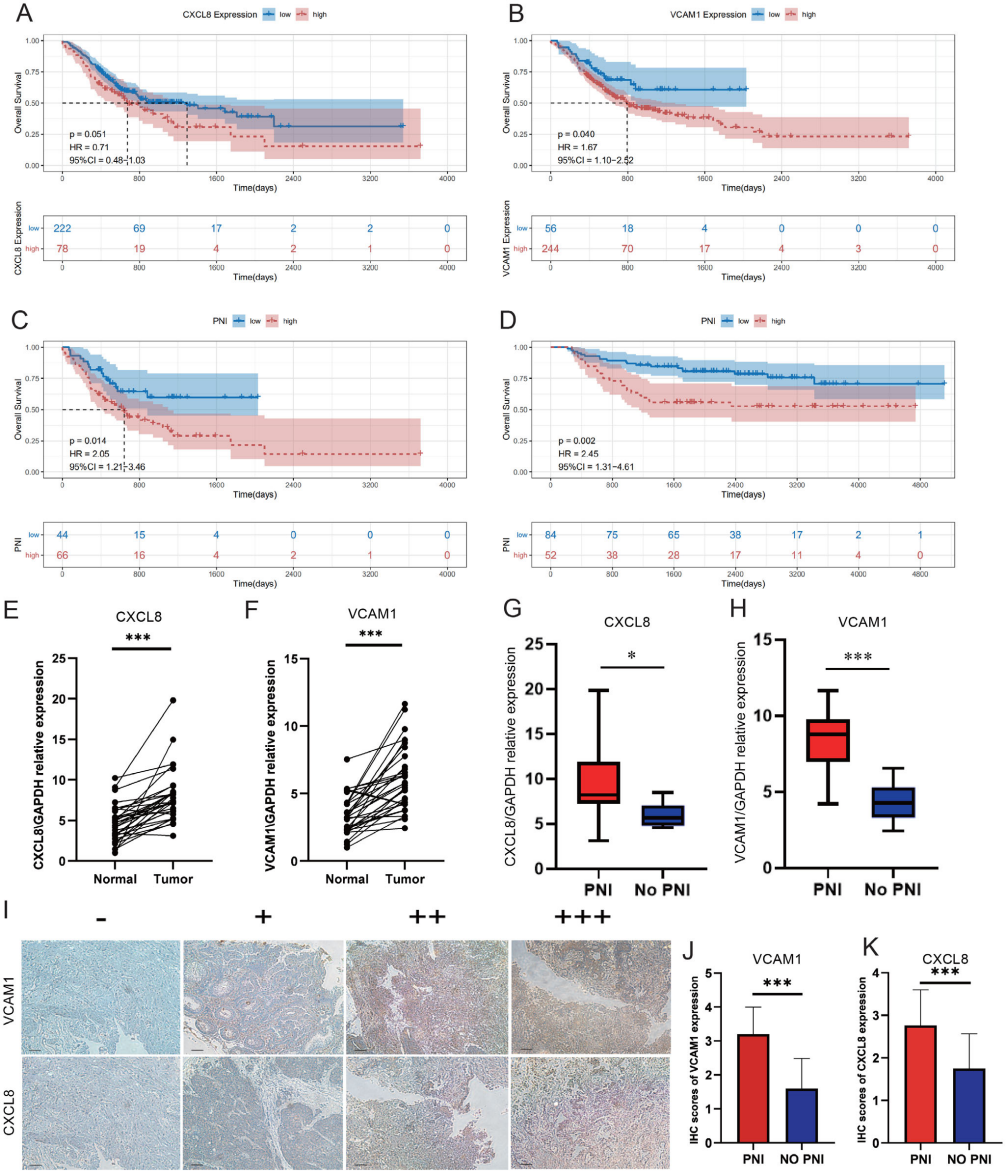
CXCL8 and VCAM1 are upregulated in gastric cancer tissues and correlate with poor clinical outcome. (A) Kaplan–Meier OS for patients with high versus low CXCL8 expression in the TCGA‐STAD cohort (log‐rank test). (B) Kaplan–Meier OS for patients with high versus low VCAM1 expression in the TCGA‐STAD cohort (log‐rank test). (C) Kaplan–Meier OS comparing tumors with concurrent high CXCL8 and high VCAM1 expression (double‐high) versus double‐low in TCGA‐STAD (log‐rank test). (D) Kaplan–Meier OS for double‐high versus double‐low CXCL8/VCAM1 expression groups in the GSE62254 cohort (log‐rank test). (E–F) Paired comparison of CXCL8 (E) and VCAM1 (F) RNA expression in tumor versus matched normal tissue (paired Wilcoxon signed‐rank test, *P < 0.05, **P < 0.01, ***P < 0.001). (G–H) Comparison of CXCL8 (G) and VCAM1 (H) RNA expression between PNI+ and PNI‐ tumor samples (Wilcoxon rank‐sum test); *P < 0.05, **P < 0.01, ***P < 0.001. (I) Immunohistochemistry (IHC) scoring distribution for CXCL8 and VCAM1 in tumor specimens (“–” = negative, “+” = low, “++” = moderate, “+++” = high). Representative images shown in main panels. (J,K) Quantification of IHC expression (PNI vs NO‐PNI) for VCAM1 (J) and CXCL8 (K). Group comparisons were performed by chi‐square test or Fisher's exact test as appropriate; *P < 0.05, **P < 0.01, ***P < 0.001.

In the TCGA‐STAD study group, we divided the patients into high‐expression and low‐expression groups based on the optimal cut‐off value of each gene, and conducted Kaplan‐Meier survival analysis. Tumours with high CXCL8 expression exhibited poorer overall survival compared with the low‐expression group (log‐rank P = 0.051; Figure 3A), and a similar trend was observed for VCAM1 (log‐rank P = 0.040; Figure 3B). Meanwhile, patients with high levels of both CXCL8 and VCAM1 had significantly lower survival rates compared to the group with low expression of both (log‐rank test P = 0.014; Figure 3C). This finding was independently validated in the GSE62254 cohort, where the same grouping strategy yielded consistent results (log‐rank P = 0.002; Figure 3D), reinforcing the robustness of combined CXCL8 and VCAM1 expression as a prognostic marker in PNI‐positive patients. Continuous variable univariate Cox regression analysis(Table S2) revealed that high VCAM1 expression was significantly associated with shorter overall survival (P < 0.05). For CXCL8, no significant linear association with prognosis was observed in continuous Cox regression. Notably, the core prognostic signature identified in this study is the concurrent high expression of VCAM1 and CXCL8, which strongly predicts poor outcome especially in PNI‐positive gastric cancer patients. The lack of linear correlation for CXCL8 alone may be attributed to its context‐dependent synergistic effect with VCAM1, as well as the mixed cellular origin of CXCL8 expression in bulk tumor tissues.

To validate these cohort‐level observations at the tissue level, we examined paired post‐surgical samples (tumour versus adjacent normal) from GC patients. Quantitative analyses demonstrated that CXCL8 and VCAM1 mRNA levels were significantly higher in tumours than in matched normal tissues (CXCL8: P < 0.001, Figure 3E; VCAM1: P < 0.001, Figure 3F). Furthermore, when tumours were stratified by PNI status, both CXCL8 and VCAM1 mRNA levels were significantly elevated in PNI‐positive tumors compared with PNI‐negative tumors (CXCL8: P < 0.05; VCAM1: P < 0.001; Figure 3G, H).

At the same time, we performed immunohistochemical staining on the surgical specimen tissue sections and conducted semi‐quantitative analysis using the H‐score. The results were compared between the PNI‐positive group and the PNI‐negative group. CXCL8 and VCAM1 staining intensities were significantly greater in PNI‐positive tumours (H‐score: CXCL8, P < 0.001; VCAM1, P < 0.001; Figure 3I–K).

We further performed stratified analyses in the TCGA‐STAD cohort to explore the associations between CXCL8, VCAM1, MIF expression and clinicopathological characteristics (Tables S3–S5). High VCAM1 expression was significantly associated with advanced T stage (T3‐T4), lymph node metastasis (N2‐N3), distant metastasis (M1), advanced pStage (III‐IV), diffuse‐type Lauren classification, and positive perineural invasion (all P < 0.05). High CXCL8 expression was significantly associated with male gender, diffuse‐type Lauren subtype, and positive PNI (P < 0.05). High MIF expression was significantly associated with advanced T stage, diffuse‐type Lauren subtype, pathological type, and positive PNI (P < 0.05).

We further investigated the association between the MIF‐VCAM1‐CXCL8 axis and the tumor microenvironment (TME) in the TCGA‐STAD cohort(Figures S1–S3). Patients were stratified into high‐ and low‐expression groups based on mRNA levels of MIF, VCAM1, and CXCL8, and the proportions of TME infiltrating cell types were compared. High VCAM1 expression was accompanied by significantly increased infiltration of cancer‐associated fibroblasts (CAFs), endothelial cells, macrophages, pericytes, and plasma cells (all P < 0.05). High CXCL8 expression was associated with elevated inflammatory monocytes and mast cells, but decreased CD8^+^ T cell infiltration (all P < 0.05). In contrast, MIF expression showed no significant correlation with TME cell composition. These results indicate that the VCAM1‐CXCL8 signaling arm shapes a pro‐tumorigenic, stromal‐rich, and immunosuppressive microenvironment that facilitates perineural invasion in gastric cancer.

These cohort and tissue‐level data concordantly identify CXCL8 and VCAM1 as upregulated in gastric tumours, enriched in PNI‐positive cases, and associated with adverse clinical outcome.

### 3.4 Tumour‐Derived CXCL8 Activates Neuronal CXCR2 to Induce MIF Expression and Secretion

**FIGURE 4:**
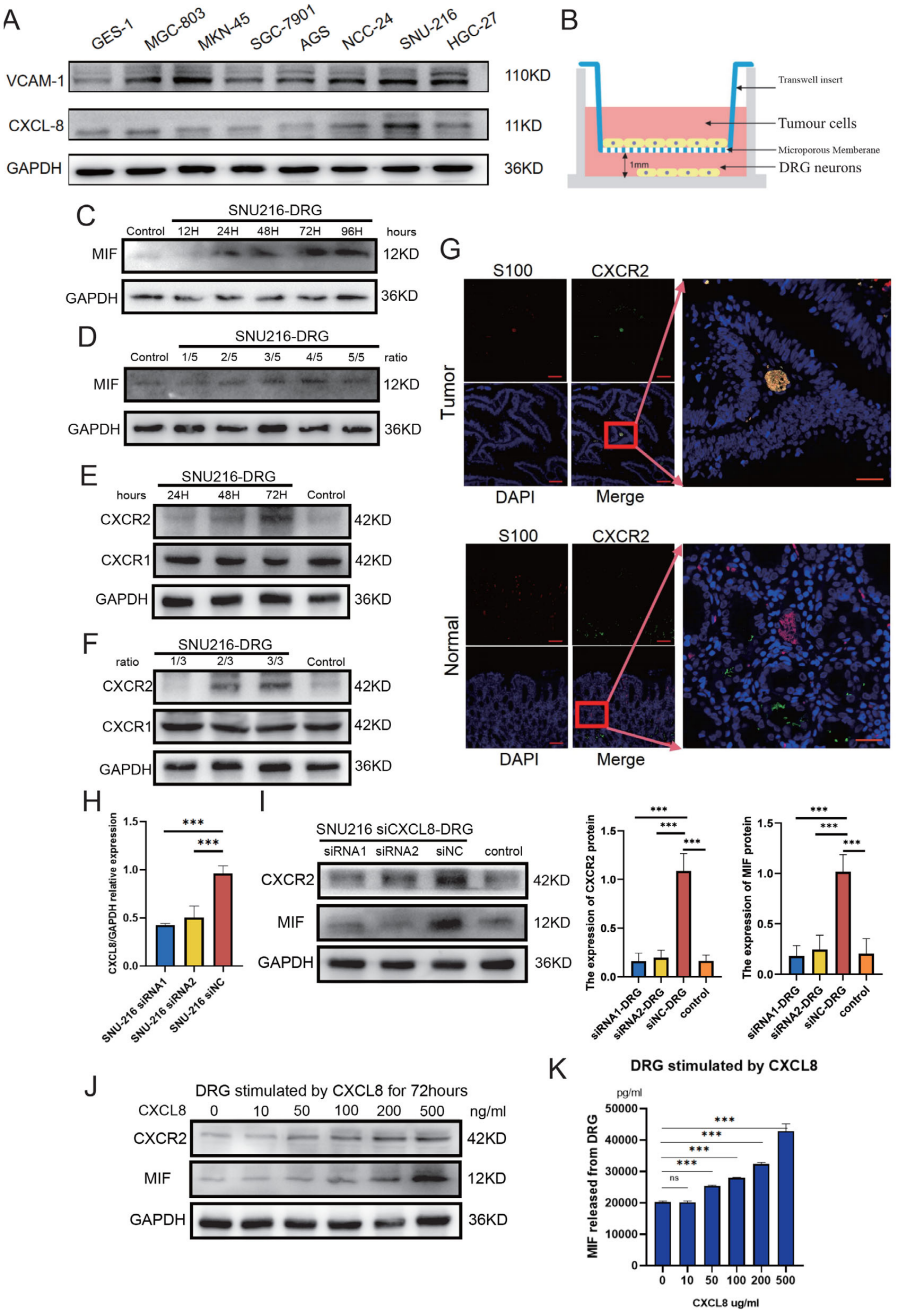
Tumour‐derived CXCL8 activates neuronal CXCR2 to induce MIF expression and secretion. (A) Representative Western blots of VCAM1 and CXCL8 protein in multiple gastric cancer cell lines and the immortalized normal gastric epithelial cell line GES‐1. (B) Schematic of the Transwell tumor–nerve co‐culture system: dorsal root ganglion (DRG) neurons in the lower chamber, tumor cells in the upper chamber. (C) Time‐dependent MIF expression in DRG neurons following co‐culture with tumor cells (Western blot). Without co‐culture as control group. (D) MIF expression in DRG neurons after 72 h of co‐culture at varying tumor:neuron ratios (Western blot). Without co‐culture as control group. (E,F) CXCR1 and CXCR2 expression in DRG neurons across co‐culture durations (E) and across tumor ratio stimulations (F) (Western blot). Without co‐culture as control group. (G) Immunofluorescence of tumor and paired normal tissues stained for S100 (neural marker, Alexa Fluor‐647, red) and CXCR2 (Alexa Fluor‐488, green); nuclei counterstained with DAPI (blue). Merged images shown. Low‐resolution images (4×) and high‐resolution images (20×) are displayed; scale bars are indicated in panels. (H) qRT‐PCR quantification following siRNA knockdown in SNU‐216 (H) (knockdown efficiency; mean ± SD). (I) Western blot and quantification of CXCR2 and MIF protein levels in DRG neurons after co‐culture with siRNA‐treated SNU‐216 cells (mean ± SD). Without co‐culture as control group. (J) Western blot of CXCR2 and MIF in DRG neurons stimulated with graded concentrations of protein CXCL8. (K) ELISA quantification of MIF in co‐culture supernatants following graded CXCL8 stimulation (mean ± SD). Statistical comparisons were performed by Student's t‐test or ANOVA with post hoc tests as indicated; *P < 0.05, **P < 0.01, ***P < 0.001.

To construct an in vitro model for mechanistic validation, we first profiled VCAM1 and CXCL8 protein expression across a panel of gastric cancer cell lines (MGC‐803, MKN‐45, SGC‐7901, AGS, NCC‐24, SNU‐216, and HGC‐27) and the normal gastric epithelial cell line GES‐1. Compared with GES‐1, VCAM1 expression was markedly elevated in MGC‐803, MKN‐45, NCC‐24, SNU‐216, and HGC‐27, while CXCL8 expression was particularly abundant in NCC‐24 and SNU‐216 (Figure 4A). Based on these expression profiles, we selected SNU‐216 and NCC‐24 as representative tumour models for subsequent mechanistic studies.

To confirm the purity of our primary DRG neuronal cultures and verify the neuronal origin of MIF, we performed two complementary immunofluorescence experiments. First, Tuj1 staining on three independent primary DRG cultures confirmed that nearly all cells were Tuj1‐positive neurons, demonstrating extremely high neuronal purity (Figure S9A). This ensures that no significant contamination by non‐neuronal cells (such as glial cells or fibroblasts) was introduced during subsequent co‐culture experiments with gastric cancer cells, eliminating potential confounding effects from other cell types on our results. Second, double immunofluorescence staining further showed that MIF was predominantly expressed in the cytoplasm of Tuj1‐positive neurons (Figure S9B), providing direct visual evidence that MIF is specifically localized to DRG neurons rather than contaminating non‐neuronal cells such as glial cells. Together, these results rigorously confirm that the MIF detected in our co‐culture system is neuron‐derived, supporting the core mechanism of our proposed signaling loop.

In a Transwell co‐culture system (tumor cells in the upper chamber; dorsal root ganglion(DRG) neurons in the lower chamber; schematic in Figure 4B), tumour cells induced time‐ and dose‐dependent stimulation of DRG neurons: MIF protein levels in DRG progressively increased with prolonged co‐culture duration and higher tumour‐to‐neuron ratios (Figure 4C, D). Considering that the main receptors of CXCL8 are CXCR1 and CXCR2 [21], we conducted tests for CXCR1 and CXCR2 in DRG nerve cells under co‐culture conditions. Receptor profiling revealed no consistent change in CXCR1 across conditions, whereas CXCR2 was significantly upregulated in DRG under identical time‐ and dose‐dependent regimes (Figure 4E, F), implicating CXCR2 as the principal neuronal receptor mediating CXCL8 signalling. Consistently, immunofluorescence staining of paired gastric cancer samples demonstrated stronger CXCR2 signals in nerve fibers located within PNI regions compared with the nerve in normal tissues (S100‐labelled nerves, Figure 4G).

To test the necessity and sufficiency of CXCL8 in this process, we employed both gene silencing and protein stimulation strategies. Tumor cells transfected with CXCL8 siRNA (Figure 4H) elicited markedly attenuated induction of neuronal CXCR2 and MIF upon co‐culture (Figure 4I), demonstrating that tumor CXCL8 is necessary for neuronal activation in this system. On the contrary, when the dorsal root ganglion neurons were directly stimulated with the protein CXCL8, the CXCR2 and MIF in the DRG showed a dose‐dependent increase in response to CXCL8 (Western blotting; Figure 4J). We also examined the MIF in the culture medium of DRG (ELISA; Figure 4K), and the trend of MIF detection in the culture medium was similar to the result in Figure 4J.

The above research results indicate that the CXCL8 secreted by tumors promotes the synthesis and release of MIF by acting on CXCR2 in DRG neurons.

### 3.5 Neuron‐Derived MIF Feeds Back to Enhance CXCL8/VCAM1 Expression via the VCAM1–ERK/STAT3 Axis

**FIGURE 5:**
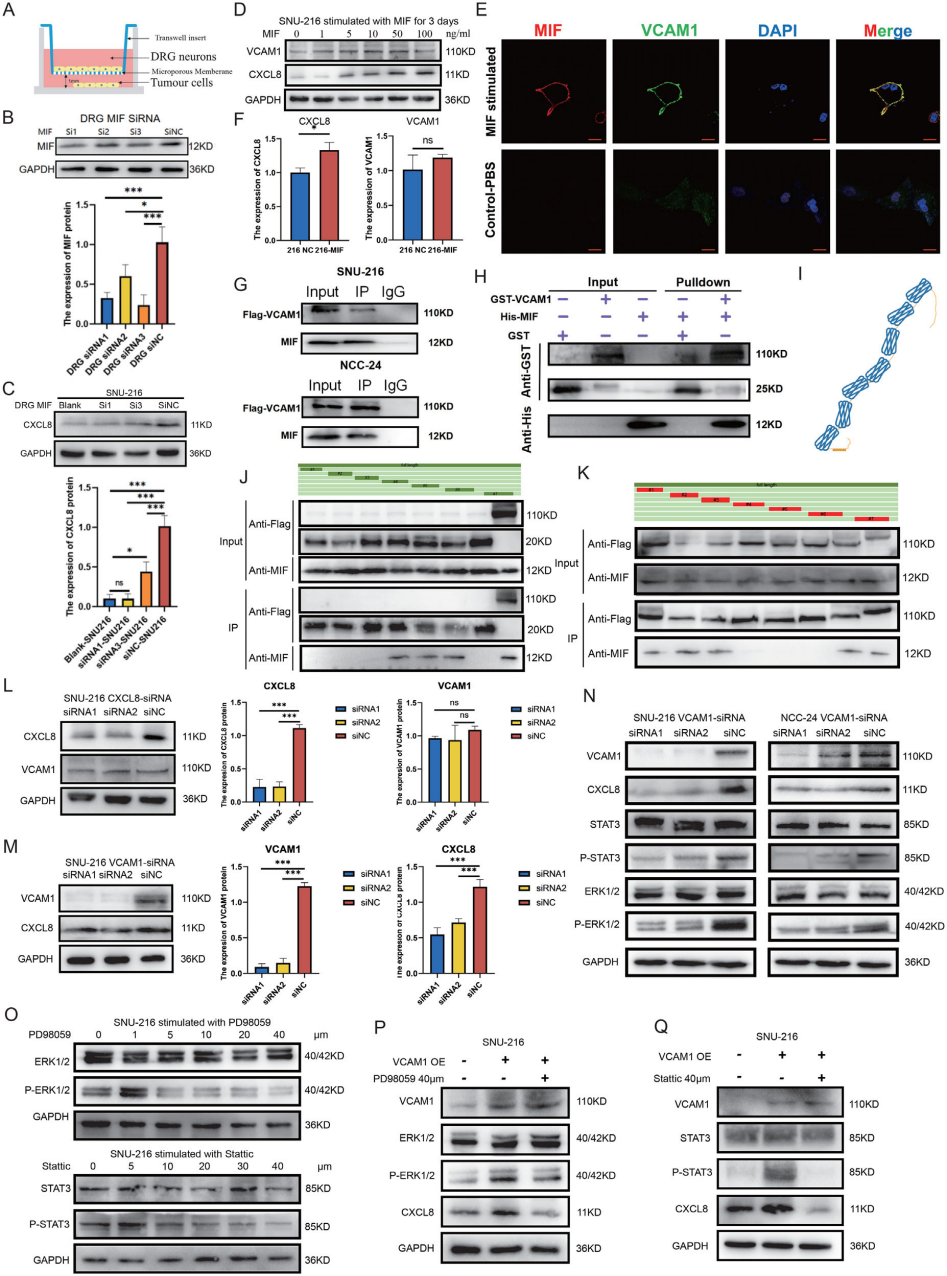
Neuron‐derived MIF feeds back to enhance CXCL8/VCAM1 expression via the VCAM1–ERK/STAT3 axis. (A) Schematic of the Transwell tumor–nerve co‐culture system: dorsal root ganglion (DRG) neurons in the upper chamber, tumor cells in the lower chamber. (B) Above is the western blot of DRG neurons after MIF siRNA knockdown. Below is the quantitative statistical analysis of the western blot results (mean ± SD). (C) Above is the western blot of SNU‐216 tumor cells after co‐culture with MIF‐silenced DRG neurons. Without DRG co‐culture as a blank group. Below is the quantitative statistical analysis of the western blot results (mean ± SD). (D) Dose‐response Western blot of SNU‐216 stimulated with protein MIF. (E) Confocal immunofluorescence of SNU‐216 after protein MIF treatment (Alexa Fluor‐647 MIF, red; VCAM1 Alexa Fluor‐488, green; DAPI, blue). Representative 40× images; scale bars shown. (F) qRT‐PCR showing CXCL8 and VCAM1 RNA induction in SNU‐216 after stimulation with 50 ng/ml recombinant MIF (mean ± SD). (G) Co‐immunoprecipitation (Co‐IP) demonstrating interaction between MIF and VCAM1 in SNU‐216 and NCC‐24 (Input = positive control; IgG = negative control). (H) GST pull‐down showing direct binding between recombinant GST‐VCAM1 and His‐MIF (GST control included). (I) Schematic of VCAM1 domain architecture derived from UniProt annotation. (J) The results of western blot showing the Co‐IP reaction between a single protein fragment constructed based on the functional domain of VCAM1 and MIF. The bar chart above shows the sequence numbers of a single fragment and the schematic representation of the full length of the protein. (K) A truncated VCAM1 protein with a single protein fragment deletion was constructed based on the functional domain of VCAM1, and the Co‐IP reaction results with MIF were obtained through western blotting. The bar chart above shows the truncated VCAM1 protein numbers without the corresponding single fragments, as well as the schematic representation of the full length of the protein. (L) Representative Western blots and knockdown quantifications for CXCL8 siRNA in SNU‐216 (demonstrating knockdown efficiency and downstream signaling changes). (M) Representative Western blots and knockdown quantifications for VCAM1 siRNA in SNU‐216 (demonstrating knockdown efficiency and downstream signaling changes). (N) The western blot results of downstream pathway changes after treating SNU‐216 and NCC‐24 cells with VCAM1 siRNA. (O) Determination of effective working concentrations for ERK phosphorylation inhibitor PD98059 and STAT3 phosphorylation inhibitor Stattic in SNU‐216. (P–Q) Western blot assay for detecting the expression changes of CXCL8 after overexpression of VCAM1 treated with PD98059 (P) or Stattic (Q) in SNU‐216. Statistical analyses used Student's t‐test or one‐way ANOVA with correction as appropriate; *P < 0.05, **P < 0.01, ***P < 0.001.

To investigate the feedback effects of neuron‐derived MIF on tumour cells, we established a Transwell co‐culture system in which DRG neurons were seeded in the upper chamber and tumour cells in the lower chamber (Figure 5A), enabling collection and analysis of tumour cell responses. We designed multiple MIF‐targeting siRNAs and identified siRNA1 and siRNA3 as the most effective (knockdown efficiency shown in Figure 5B). We conducted co‐culture experiments of DRG treated with MIF siRNA and SNU‐216. Compared with other groups, the CXCL8 level in SNU‐216 cells co‐cultured with the siNC control group increased the most significantly. During co‐culture with siMIF DRG, the expression of CXCL8 in SNU‐216 decreased, but it was higher than that in the Blank group without co‐culture (Figure 5C). This indicates that MIF in DRG neurons significantly promotes the expression of CXCL8 in tumor cells.

To further confirm that neuronal CXCR2 is the key node mediating this paracrine signaling loop, we pretreated DRG neurons with SB225002 or DMSO control, thoroughly washed away all unbound drug to exclude direct effects on tumor cells, and then co‐cultured the pretreated neurons with SNU‐216 cells. Western blot analysis showed that co‐culture with DMSO‐pretreated DRG neurons significantly upregulated both VCAM1 and CXCL8 expression in SNU‐216 cells compared with the control group (P < 0.001). In contrast, co‐culture with SB225002‐pretreated DRG neurons abrogated this upregulation (P < 0.001, Figure S4). These results provide definitive evidence that neuronal CXCR2 activation is an absolute requirement for the DRG‐induced amplification of the MIF‐VCAM1‐CXCL8 signaling axis in gastric cancer cells.

Furthermore, we observed the responses of tumor cells by stimulating them with MIF at gradient concentrations. Protein MIF induced dose‐dependent increases in CXCL8 and VCAM1 protein levels (Figure 5D), with 50 ng·ml^−^^1^ producing the most pronounced response. The results of qPCR showed that 50 ng·ml^−^^1^ MIF significantly elevated CXCL8 mRNA but did not alter VCAM1 transcript levels (Figure 5F), implying that MIF induction of CXCL8 occurs, at least in part, at the transcriptional level, whereas regulation of VCAM1 may be post‐transcriptional (e.g., translational control, membrane trafficking, or protein stability). Confocal immunofluorescence microscopy examination revealed that in the stimulated cells, MIF and VCAM1 on the cell membrane surface showed a significant co‐localization phenomenon, suggesting that MIF may have an interaction relationship with VCAM1. Moreover, after MIF treatment, the signal intensity of VCAM1 on the cell membrane surface of SNU‐216 generally increased (Figure 5E), which was consistent with the results in Figure 5D.

To confirm the relationship between VCAM1 and MIF, we first conducted a co‐immunoprecipitation (Co‐IP) experiment in tumor cells. The results showed that MIF was mainly present in the immunoprecipitate of VCAM1‐FLAG (Figure 5G), indicating a direct or indirect interaction between VCAM1 and MIF. Therefore, we further conducted a GST‐pulldown experiment of MIF and VCAM1. GST‐pulldown result (GST‐VCAM1 / His‐MIF) further demonstrated direct VCAM1‐MIF binding (Figure 5H). According to the protein functional domains classification of VCAM1 (UniProt), VCAM1 mainly consists of 7 protein fragments of functional domains (Figure 5I). Based on these 7 functional domain protein fragments, we separately constructed a single protein fragment (Figure 5J) and a truncated VCAM1 protein (Figure 5K), and then conducted Co‐IP experiments with MIF. The results of Co‐IP analysis indicated that the functional domain protein fragments numbered 4, 5, and 6 in VCAM1 were the regions directly bound by MIF.

We next examined the regulatory relationship between VCAM1 and CXCL8 and their downstream pathways in tumour cells. Western blotting revealed that silencing CXCL8 had no effect on VCAM1 levels (Figure 5L), whereas silencing VCAM1 significantly reduced CXCL8 expression (Figure 5M). This indicates that VCAM1 regulates the expression of CXCL8. Given that there was previously evidence suggesting an association between CXCL8 and STAT3 and ERK [28], we then tested whether VCAM1 would affect these pathways. In fact, by knocking down the VCAM1 gene, the levels of p‐STAT3 and p‐ERK were significantly reduced, and the expression of CXCL8 was also decreased (Figure 5N). This indicates that VCAM1 regulates the expression of CXCL8 by influencing the phosphorylation levels of STAT3 and ERK. We treated SNU‐216 with gradient concentrations of the ERK phosphorylation inhibitor (PD98059) and the STAT3 phosphorylation inhibitor (Stattic). By detecting the phosphorylated proteins of ERK and STAT3, it was found that under the action of 40 µM PD98059 and Stattic, the phosphorylation levels of ERK and STAT3 in SNU‐216 were well inhibited (Figure 5O). Therefore, we chose this concentration of PD98059 and Stattic for the experiment. In SNU‐216, we performed overexpression(OE) of VCAM1 and then used PD98059 and Stattic to inhibit the phosphorylation of ERK and STAT3, in order to observe the expression of CXCL8. In Figure 5P,Q, overexpression of VCAM1 would promote the expression of CXCL8, but after the inhibition of ERK and STAT3 phosphorylation, the expression of CXCL8 would decrease. These results demonstrate that VCAM1 regulates the expression of CXCL8 by influencing the phosphorylation of ERK and STAT3.

To verify whether VCAM1 is required for MIF‐mediated downstream signaling, rescue experiments were performed(Figure S5). VCAM1 was stably silenced in gastric cancer cells, which were then treated with recombinant MIF protein. MIF‐induced phosphorylation of ERK and STAT3, as well as upregulation of CXCL8, were markedly reversed by VCAM1 knockdown. These results indicate that VCAM1 acts as a functional receptor essential for MIF‐triggered ERK/STAT3 activation and CXCL8 expression in gastric cancer cells.

Overall, these in vitro experiments revealed a functional reaction chain: the MIF secreted by neurons interacts with the VCAM1 on tumor cells, activates the ERK/STAT3 signaling pathway, and induces the expression of CXCL8. Subsequently, CXCL8 acts on CXCR2 on neurons, further promoting the expression of MIF on neurons, forming a positive feedback loop (MIF‐VCAM1‐CXCL8), thereby strengthening the interaction between neurons and tumors.

### 3.6 The MIF‐VCAM1‐CXCL8 Axis can Drive the Migration, Invasion and Proliferation of Tumor Cells

**FIGURE 6:**
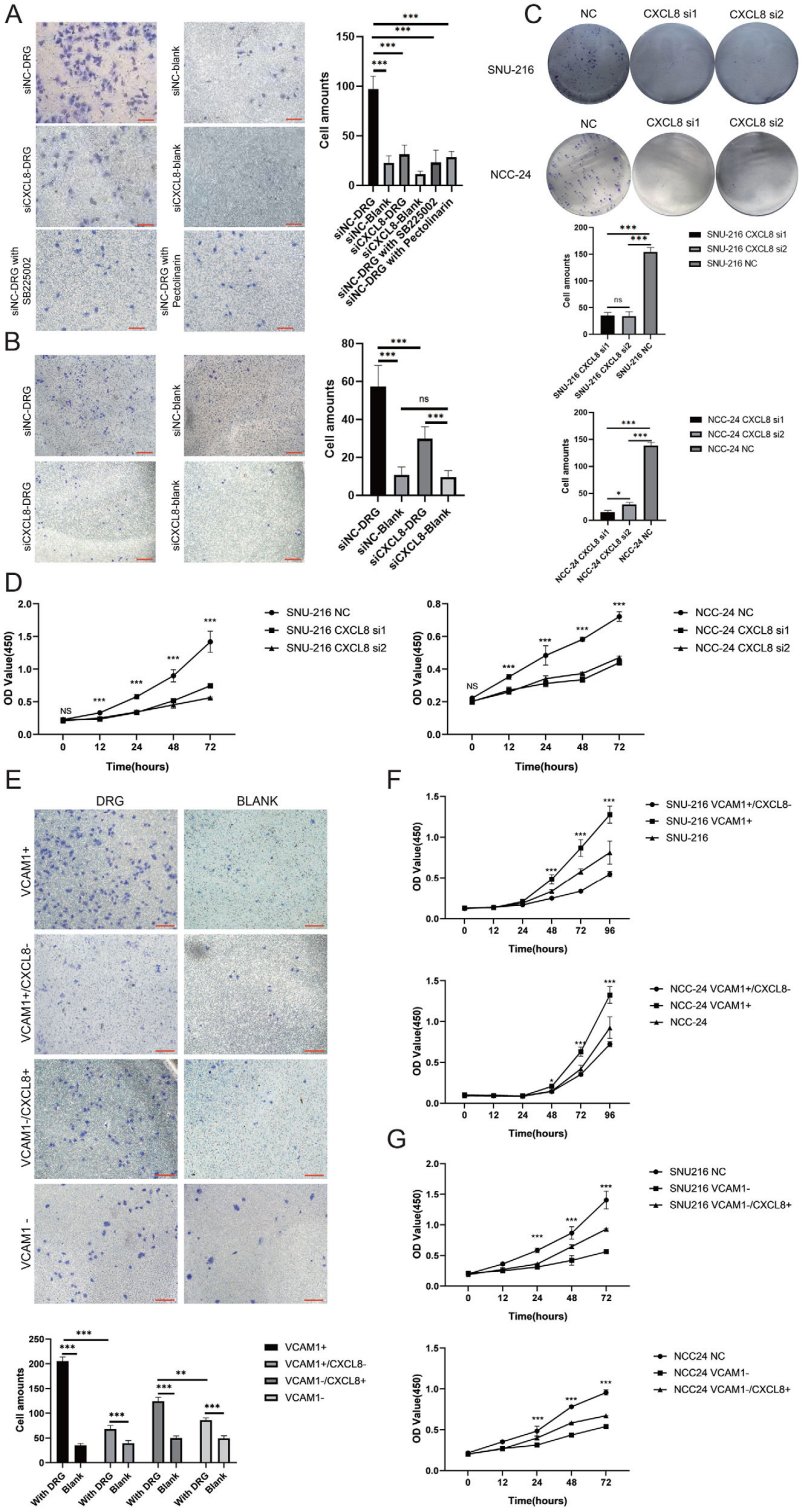
The MIF‐VCAM1‐CXCL8 axis can drive the migration, invasion and proliferation of tumor cells. Representative images of Transwell tumor cell migration assays in the co‐culture model. DRG: lower chamber containing DRG neurons; Blank: lower chamber without neurons; SB225002(inhibiting the binding of CXCL8 and CXCR2) and Pectolinarin(inhibiting the production of CXCL8) indicate pharmacologic treatment groups (DRG present). On the right is the quantification of migration assay results (mean ± SD; cell counts in random fields). (B) Representative Transwell invasion assay images (Matrigel‐coated inserts) comparing DRG, siCXCL8 and siNC groups. DRG: lower chamber containing DRG neurons; Blank: lower chamber without neurons. On the right is the quantification of invasion assay results (mean ± SD; cell counts in random fields). (C) Representative colony formation images for SNU‐216 and NCC‐24 after CXCL8 knockdown. Below is the quantitative statistical result of the clone formation experiment (mean ± SD). (D) CCK‐8 proliferation curves of SNU‐216 and NCC‐24 following cxcl8 siRNA treatment (mean ± SD). (E) Representative images and quantification of migration recovery assays for SNU‐216 (DRG present versus blank controls; mean ± SD). Specifically, after overexpression of VCAM1, siCXCL8 treatment was performed, and after interference with VCAM1 expression, CXCL8 overexpression was carried out. (F, G) CCK‐8 recovery experiments of SNU‐216 and NCC‐24 (proliferation rescue following intervention; mean ± SD). After overexpression of VCAM1, siRNA interference was performed on the downstream CXCL8. And after interfering with VCAM1, overexpression of CXCL8 was carried out as a downstream treatment. Statistical comparisons were performed using Student's t‐test, one‐way ANOVA or nonparametric equivalents as appropriate. For categorical data, chi‐square or Fisher's exact tests were used. *P < 0.05, **P < 0.01, ***P < 0.001.

To evaluate the biological role of the MIF‐VCAM1‐CXCL8 axis in tumour progression, we first performed a series of functional assays using an in vitro Transwell neuron‐tumour co‐culture system. In the tumor migration experiment, based on the neuro‐tumor co‐culture model, we conducted experiments by interfering with CXCL8 in tumor cells and using SB225002 (an inhibitor of CXCL8 and CXCR2 binding) and Pectolinarin (an inhibitor of CXCL8 release) as intervention conditions. As shown in Figure 6A, compared with the blank control group, nerve cells significantly enhance the migration ability of tumor cells; while after interfering with the expression of CXCL8 in tumor cells, the migration ability of the tumor is significantly inhibited. At the same time, similar effects were observed in the SB225002 group and the Pectolinarin group, where inhibiting the binding of CXCL8 and CXCR2 and inhibiting the expression of CXCL8 in tumor cells both significantly inhibit the migration ability of tumor cells. In the neuro‐tumor co‐culture model, we also verified the tumor invasion ability. The results are shown in Figure 6B. Compared with the blank control group, the nerve cells significantly enhanced the invasion ability of the tumor cells. After interfering with the expression of CXCL8 in the tumor cells, the invasion ability of the tumor cells was significantly inhibited.

In proliferation assays, both colony formation and CCK‐8 assays consistently demonstrated that CXCL8 knockdown significantly reduced tumour cell clonogenicity and proliferation rate (Figure 6C, D; colony counts/OD values: siNC vs siCXCL8, P < 0.001). Based on the relationship between VCAM1 and CXCL8 as verified in Figure 5, we conducted rescue and re‐expression experiments. Overexpression of VCAM1 promoted migration and proliferation, whereas concomitant CXCL8 silencing significantly attenuated these effects. Conversely, overexpression of CXCL8 in VCAM1‐silenced cells partially restored migratory and proliferative capacity (Figure 6E–G).

### 3.7 The MIF‐VCAM1‐CXCL8 Axis can Promote the Proliferation and Neuroinvasion of Tumor Cells in Vivo

**FIGURE 7:**
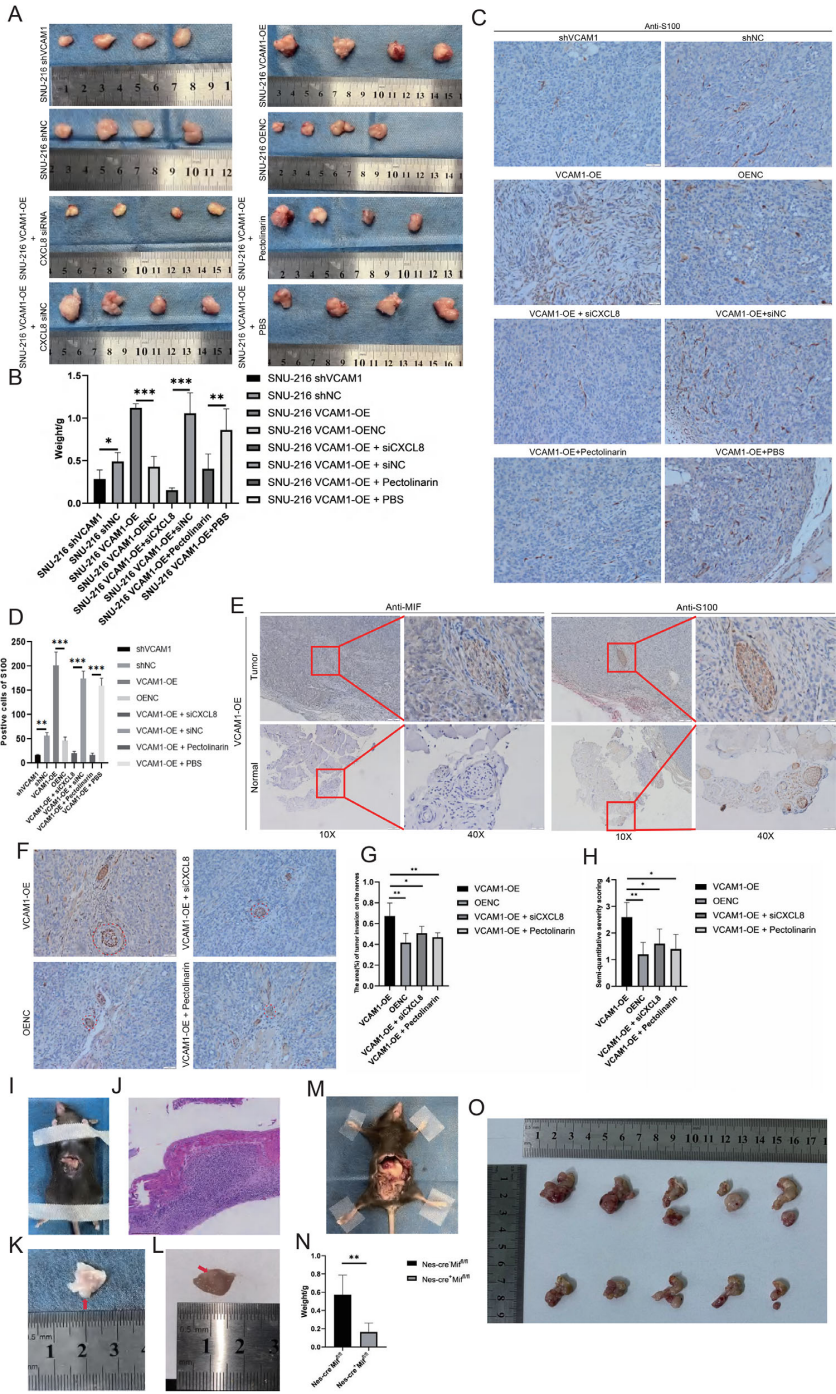
The MIF‐VCAM1‐CXCL8 axis can promote the proliferation and neuroinvasion of tumor cells in vivo. (A) In the simulated model of nerve invasion involving sciatic nerve tumors, the anatomical images of the tumors. The transplanted gastric cancer cell line used for inoculation was SNU‐216. SNU‐216 was subjected to VCAM1 interference, overexpression, and corresponding control treatments respectively. Based on the overexpression of VCAM1, intratumoral siRNA injection of downstream CXCL8 (with siNC as the control) was performed, as well as intraperitoneal injection of Pectolinarin (to inhibit the production of CXCL8, with PBS as its control). (B) Statistical analysis of the quality of transplanted tumors in different groups (mean ± SD, n = 4 mice per group). (C) Representative IHC images assessing density of nerve fibers in tumors by S100 staining (DAB brown; hematoxylin counterstain), 20× magnification. (D) Quantification of nerve fibers density across groups (mean ± SD). (E) Representative comparative IHC images for MIF and S100 inside versus outside tumor regions. (F) Representative S100 IHC images for assessing PNI in sciatic nerve invasion mice (DAB brown; hematoxylin counterstain), 20× magnification. (G) Statistical analysis of the percentage of nerve fascicle area invaded by tumor cells among groups (n = 4, mean ± SD). (H) Statistical analysis of semi‐quantitative severity scoring for PNI among groups (n = 4, mean ± SD). (I–L) Representative images and histological images of the in situ spontaneous gastric cancer model generated by the “Sleeping Beauty” transposon system: (I) Gastric tumor occurrence in mice (2 weeks after injection of the plasmid mixture), (J) H&E staining images of the tumor 2 weeks after plasmid mixture injection, (K, L) Serosal(protruding tumor, with red arrow) and mucosal(volcano‐shaped ulcer, with red arrow) appearances at 2 weeks. (J) In the C57BL/6 Nes‐Cre^−^ Mif^fl/fl^ and C57BL/6 Nes‐Cre^+^ Mif^fl/fl^ groups, mixed plasmid injection was used to induce the occurrence of gastric cancer in mice. Representative images of gastric cancer in mice at the end of the 4‐week termination experiment. (K) Comparison of tumor weights between C57BL/6 Nes‐Cre^−^ Mif^fl/fl^ (control group) and C57BL/6 Nes‐Cre^+^ Mif^fl/fl^ (conditional knockout) mice (mean ± SD, n = 5 mice per group). (L) Representative ex vivo stomach images from control and conditional knockout animals; red arrows indicate abdominal/pelvic metastases. Statistical comparisons were performed using Student's t‐test, one‐way ANOVA or nonparametric equivalents as appropriate. For categorical data, chi‐square or Fisher's exact tests were used. *P < 0.05, **P < 0.01, ***P < 0.001.

To validate these mechanisms in vivo and assess therapeutic potential, we employed a modified sciatic nerve invasion xenograft model in nude mice (adapted from Sylvie Deborde et al.) [27]. Stable VCAM1‐overexpressing (OE) or VCAM1‐silenced (shVCAM1) gastric cancer cells(SNU‐216) were implanted(n = 4 mice per group). Compared with shNC controls, shVCAM1 tumours displayed significantly reduced tumour weight (mean ± SD, P < 0.05), whereas VCAM1‐OE tumours grew substantially larger (OE vs. OENC, P < 0.001) (Figure 7A, B). Importantly, intra‐tumoural injection of siCXCL8 or intraperitoneal injection of Pectolinarin attenuated the tumour‐promoting effect of VCAM1 overexpression (Figure 7A, B; siCXCL8 vs. siNC, P < 0.001; Pectolinarin vs. PBS control, P < 0.01), suggesting that targeting CXCL8 can suppress VCAM1‐driven tumour growth in vivo.

We conducted a statistical analysis of the density of nerve fibers within the tumors. Histological analysis (S100 IHC) revealed increased nerve density and invasion in VCAM1‐OE tumours compared with OENC, whereas shVCAM1, siCXCL8, or Pectolinarin treatment significantly reduced the degree of perineural invasion (Figure 7C, D). Serial sections demonstrated stronger MIF staining within nerves infiltrated by VCAM1‐OE tumours, whereas uninvolved extra‐tumoural nerves showed no significant change. Positive staining was most prominent in neuronal cell bodies, indicating a neuronal origin of MIF (Figure 7E). Consistent with the PNI phenotype of human gastric cancer, this animal model exhibited highly similar neural invasion behaviors (Figure 7F). As shown in Figure 7G, compared with the OENC group, the VCAM1‐OE group had a significantly higher percentage of tumor‐invaded area relative to the total cross‐sectional area of the nerve fascicle. Importantly, both siCXCL8 intervention and Pectolinarin treatment significantly reversed the increase in nerve invasion area induced by VCAM1 overexpression. Semi‐quantitative PNI severity scoring (Figure 7H) showed consistent results: VCAM1 overexpression significantly increased the PNI severity score, while treatment with siCXCL8 or Pectolinarin significantly reduced the PNI grade.

To genetically validate the role of neuron‐derived MIF, we generated neuron‐specific Mif conditional knockout mice (C57BL/6‐Nes‐cre^+^ Mif^fl/fl^), with genotyping confirmed by PCR (Mif^fl/fl^, 405 bp; wild‐type, 273 bp; Nes‐cre, 150 bp; Figure S6). We also verified the neuron‐specific knockout efficiency of Mif in DRG tissues. Compared with Nes‐cre^−^Mif^fl/fl^ mice, MIF expression at both mRNA and protein levels was significantly decreased in DRG tissues from Nes‐cre^+^Mif^fl/fl^ mice (Figure S7A,B). Lineage tracing using the ROSA26‐tdTomato reporter system confirmed that Cre recombinase activity was strictly restricted to Nestin‐positive neurons in gastric tissues, with no detectable tdTomato signal in non‐neuronal cells (Figure S7C). These results confirmed that Mif was specifically and efficiently knocked out in neurons, validating the reliability of the mouse model. In the context of Nestin‐Cre‐mediated neuronal Mif deletion, mice developed spontaneous gastric cancer using an AKT/MYC Sleeping Beauty transposon system. We dissected the stomach of the mice (2 weeks after injection of the plasmid mixture) and could observe characteristic protrusions on the serosal surface, and typical crater‐like ulcers on the mucosal surface (Figure 7I,K,L). Histological examination confirmed that this spontaneous tumor exhibited typical characteristics of invasion from the mucosa to the serosa (Figure 7J). To further validate the clinical relevance of our in vivo models, we performed both S100 immunohistochemical staining and HE staining on human PNI‐positive gastric cancer specimens and corresponding mouse tumor tissues. As shown in Figure S8A, S100 staining revealed that the perineural invasion features in our mouse models were highly consistent with those in human gastric cancer: tumor cells infiltrated along the nerve sheath, invaded into the perineurium and endoneurium, and disrupted normal neural architecture, recapitulating the classic pathological hallmarks of human gastric cancer PNI. HE staining further demonstrated that the overall histological characteristics of mouse tumors faithfully recapitulated those of human gastric cancer, including marked cellular atypia, disordered tissue architecture, and infiltrative growth pattern(Figure S8B). We terminated the experiment after the fourth week following the injection of the mixed plasmids. By this time, significant tumor had formed on the stomach body of the mouse (Figure 7M). Tumour growth and neural invasion were markedly suppressed in knockout mice compared with controls (tumour weight: Nes‐cre^−^Mif^fl/fl^ vs Nes‐cre^+^Mif^fl/fl^, mean ± SD, P < 0.01), and some animals also exhibited abdominal wall and peritoneal metastases (Figure 7N, O; n = 5 mice per group). These results demonstrate that the absence of Mif in neurons significantly reduces the invasive and metastatic abilities of tumor cells.

Together, these in vivo findings converge to establish that the MIF–VCAM1–CXCL8 axis drives gastric cancer progression by enhancing tumour cell proliferation, migration, and perineural invasion.

## 4 Discussion

PNI stands as a critical pathological hallmark of gastric cancer, tightly linked to advanced disease stages, heightened recurrence risks, and dismal patient prognoses [2, 3, 4]. Despite growing recognition of PNI as a bidirectional crosstalk between tumor cells and nerves, the intricate molecular circuitry driving this interplay remains poorly delineated. In this study, we systematically unravel a novel MIF‐VCAM1‐CXCL8 positive‐feedback axis that orchestrates tumor‐nerve communication, shedding light on the mechanistic basis of PNI and gastric cancer progression while identifying actionable therapeutic targets.

Our integrative transcriptomic analyses of TCGA and GEO cohorts first pinpointed CXCL8 and VCAM1 as core regulators of PNI. Both molecules were robustly upregulated in PNI‐positive tumor tissues, and their concurrent high expression predicted inferior overall survival and increased distant metastasis. This clinical relevance underscores the potential of CXCL8 and VCAM1 as prognostic biomarkers, offering a means to stratify gastric cancer patients at high risk of PNI and guide personalized treatment strategies. Notably, functional enrichment analyses revealed that the gene signature associated with elevated CXCL8/VCAM1 expression was enriched in immune activation, cytokine‐cytokine receptor interactions, and secretory signaling processes, aligning with the established roles of chemokines and adhesion molecules in tumor‐stromal crosstalk [29, 30].

Mechanistically, we uncovered a sequential signaling cascade that forms the backbone of tumor‐nerve crosstalk. Tumor‐derived CXCL8 acts as an initial paracrine signal, engaging CXCR2 on DRG neurons to induce MIF expression and secretion. This finding extends prior observations of CXCL8/CXCR2 signaling in tumor progression by highlighting its role in activating neural cells—a previously underappreciated dimension of the CXCL8 pathway [31, 32, 33]. Importantly, we confirmed that CXCR2, rather than CXCR1, is the primary neuronal receptor mediating CXCL8 responses, providing a specific molecular target within the axis.

Subsequently, neuron‐secreted MIF feeds back to tumor cells by directly binding VCAM1, as validated through co‐immunoprecipitation and GST pull‐down assays. Domain mapping further identified VCAM1's 4th, 5th, and 6th functional domains as critical for MIF interaction, laying the groundwork for future structural studies to develop targeted inhibitors of this binding event. This MIF‐VCAM1 interaction activates the ERK/STAT3 signaling pathway, which in turn upregulates CXCL8 expression in tumor cells—completing a self‐amplifying positive‐feedback loop. Notably, our data suggest that MIF regulates CXCL8 at the transcriptional level while modulating VCAM1 through post‐transcriptional mechanisms (like protein stability), indicating distinct regulatory modes within the axis. This dual regulation underscores the complexity of the circuit and its potential resilience, emphasizing the value of targeting multiple nodes for therapeutic intervention.

Functional investigations, encompassing both in vitro and in vivo models, validated the biological relevance of this axis. In vitro, perturbation of CXCL8, VCAM1, or MIF compromised tumor cell proliferation, migration, and invasion—findings further supported by rescue assays, which confirmed that CXCL8 functions downstream of VCAM1 to mediate these pro‐tumorigenic phenotypes. In vivo, utilizing a sciatic nerve invasion xenograft model, we demonstrated that VCAM1 overexpression enhances tumor growth and PNI, whereas VCAM1 silencing or CXCL8 targeting—either via siRNA‐mediated knockdown or treatment with the small‐molecule inhibitor Pectolinarin—abrogated these pathogenic phenotypes. Critically, neuron‐specific MIF conditional knockout mice displayed markedly diminished tumor growth, PNI, and metastasis in an orthotopic gastric cancer model—genetically corroborating the indispensable role of neuron‐derived MIF in orchestrating the axis. Collectively, these preclinical findings solidify the MIF‐VCAM1‐CXCL8 axis as a non‐redundant driver of gastric cancer progression.

Notably, beyond simply enhancing tumor invasive activity, our findings support a potential spatial guidance role of the MIF–VCAM1–CXCL8 feedback loop during the early stages of PNI. As a classic chemokine, tumor‐derived CXCL8 initiates the attraction of gastric cancer cells toward nerve fibers. Meanwhile, CXCL8 stimulates neurons to secrete MIF, which may accumulates and forms a locally concentrated chemotactic gradient within the narrow perineural space. This spatially restricted gradient further guides and directs tumor cell movement along the nerve sheath, thereby defining the invasion path of PNI. Therefore, the MIF–VCAM1–CXCL8 axis not only functionally strengthens tumor proliferation and invasion but also provides a spatial guidance signal that orchestrates the directional invasion of tumor cells into the neural compartment. This new perspective elevates our understanding of the feedback loop from a functional amplifier to a spatial navigator in gastric cancer PNI.

Our work advances the understanding of tumor‐nerve crosstalk by integrating three key molecular players—CXCL8 (chemokine), MIF (neuron‐derived cytokine), and VCAM1 (adhesion receptor)—into a coordinated signaling module. While prior studies have implicated each individual component of this axis in cancer progression, their coordinated involvement in PNI and tumor‐nerve crosstalk has remained uncharacterized. For instance, VCAM1 has been predominantly investigated in the context of tumor‐endothelial adhesion and angiogenesis [34, 35], whereas our findings uncover a novel function for VCAM1 in mediating tumor–neuron crosstalk through direct MIF binding and subsequent ERK/STAT3 pathway activation. Likewise, MIF has been extensively associated with tumor immunity and metabolic regulation [36, 37], yet its role as a neuron‐derived signal that drives PNI represents a previously unreported mechanistic insight. Notably, classic receptors for MIF, including CD74, CXCR2, and CXCR4, have been widely reported to mediate MIF signaling in various cancers. In the present study, we identified VCAM1 as a novel functional receptor that interacts with MIF and drives PNI in gastric cancer. It is plausible that VCAM1 may cooperate with canonical MIF receptors to amplify downstream signaling, which warrants further investigation.

Clinically, our findings offer multiple therapeutic entry points. Targeting CXCL8 (like neutralizing antibodies or CXCR2 inhibitors), blocking MIF‐VCAM1 interaction (like domain‐specific peptides), or inhibiting ERK/STAT3 signaling could disrupt the positive‐feedback loop and mitigate PNI. Notably, CXCR2 inhibitors [38, 39] and ERK/STAT3 antagonists [40, 41] are already in preclinical or clinical development for other cancers, providing a translational shortcut for repurposing these agents in gastric cancer. Additionally, the prognostic value of CXCL8/VCAM1 co‐expression could be integrated into existing clinical nomograms to improve risk stratification, enabling earlier intervention for high‐risk patients.

Despite these advances, several limitations warrant consideration. First, the atomic‐level structure of the VCAM1‐MIF complex remains unresolved, which is essential for rational drug design to target this interaction. Second, while our study focuses on gastric cancer, future work should explore whether this axis is conserved in other PNI‐prone cancers (like pancreatic, prostate, or colorectal cancer) to determine its broader applicability. Third, our in vitro mechanistic studies were performed using rat neonatal DRG neurons, while in vivo validation was conducted in mouse models. This experimental design is widely adopted in the PNI field due to the technical advantages of rat DRG for in vitro culture and the availability of genetic tools in mice. Although our results are consistent across different experimental systems, potential subtle species‐specific differences cannot be completely excluded. Future studies using human iPSC‐derived neurons will help to further validate the clinical translatability of our findings. Fourth, the TCGA‐STAD/GSE62254 cohorts used for analysis consist of bulk RNA‐sequencing data, which cannot distinguish gene expression between tumor cells and tumor microenvironment cells. Future studies using single‐cell RNA‐sequencing will help to further dissect the cell‐type‐specific contributions of the MIF‐VCAM1‐CXCL8 axis to gastric cancer PNI and prognosis. Finally, the impact of the axis on the tumor immune microenvironment (an area suggested by our functional enrichment analyses) merits further investigation, as combining axis‐targeted therapies with immunotherapies could yield synergistic effects.

In conclusion, we identify and validate a MIF–VCAM1–CXCL8 positive‐feedback axis that drives PNI and gastric cancer progression by mediating bidirectional tumor–nerve crosstalk. This axis provides prognostic biomarkers (CXCL8, VCAM1) and multiple therapeutic targets (CXCL8/CXCR2, MIF–VCAM1 interaction, ERK/STAT3), offering new opportunities to improve outcomes for gastric cancer patients with PNI. Future studies will focus on developing targeted inhibitors of the axis and evaluating their efficacy in clinical trials, alongside further exploration of the axis’ role in other cancer types and its interplay with the immune system.

## Author Contributions

**Xunjun Li**: Validation, Writing – Original Draft, Writing – Review & Editing. **Zhongya Zhai**: Writing – Original Draft, Visualization. **Haiyi Yu**: Writing – Original Draft, Data Curation. **Renjie Qiu**: Writing – Review & Editing. **Fengyu Li**: Software. **Luxi Xiao**: Validation, Formal analysis. **Boxi Huang**: Software, Visualization. **Huiying Liu**: Formal analysis, Resources. **Jiayong He**: Methodology. **Bowen Cai**: Formal analysis, Methodology. **Jiawen Chen**:Software. **Jiang Yu**: Supervision. **Guoxin Li**: Investigation, Supervision. **Tao Chen**: Resources, Funding acquisition, Project administration, Writing – Review & Editing, Conceptualization.

## Funding

This work was supported by Fengshan Engineering Disciplines (2023040016), National Natural Science Foundation of China (82472066 and 82272087), Natural Science Foundation of Guangdong Province (2021B1515020055), Natural Science Foundation of Jiangxi Province (20242BAB26153), 5. Ganzhou Municipal Science and Technology Project (2022‐RC1342).

## Ethics Statement: Human | Animals

This study was conducted in accordance with the Declaration of Helsinki and Chinese ethical guidelines for clinical research. Written informed consent was obtained from all human participants, and the protocol was approved by the Medical Ethics Committee of Nanfang Hospital of Southern Medical University (FM202410270009). Animal experiments complied with the ARRIVE guidelines and were approved by the Animal Ethics Committee of Nanfang Hospital (IACUC‐LAC‐20230224‐005).

## Consent

All the authors have read and agreed to the content of the manuscript.

## Conflicts of Interest

The authors declare no conflicts of interest.

## Supporting information




**Supporting File 1**: advs76195‐sup‐0001‐SuppMat.docx.

**Supporting File 2**: advs76195‐sup‐0002‐FigureAndTableCaption.docx.

**Supporting File 3**: advs76195‐sup‐0003‐FigureS1‐S9.zip.

**Supporting File 4**: advs76195‐sup‐0004‐TableS1‐S5.zip.

## Data Availability

All the data have been presented in the figures of this article and in the supplementary materials. The experimental methods and materials have been described in detail in the manuscript. For more information, every reader can directly contact us via the email address provided.
