# Supplementary material for: Neuron‐Derived MIF Engages VCAM1 to Fuel a Self‐Amplifying CXCL8 Loop That Drives Perineural Invasion and Metastasis in Gastric Cancer
Source: Adv Sci (Weinh). 2026 Jun 22:e76195. Online ahead of print. doi: 10.1002/advs.76195 (PMC13337004; doi:10.1002/advs.76195)

Supplementary file

Methods

2.17 Bioinformatics analysis

Public transcriptomic datasets, including TCGA-STAD RNA-seq data and the GSE62254 microarray dataset, together with their corresponding clinical annotations, were retrieved from public repositories. Samples lacking key clinicopathological variables, overall survival data, or pathological perineural invasion (PNI) information were excluded from the downstream integrative analyses.

For TCGA-STAD, gene-level expression profiles were extracted and annotated according to official gene symbols. For GSE62254, probe IDs were converted to gene symbols based on the corresponding platform annotation file, and when multiple probes mapped to the same gene, the probe with the highest average expression was retained. Genes with very low expression across samples were removed before further analysis. Within each dataset, expression values were normalized separately using standard cohort-specific procedures, and expression matrices were then harmonized at the shared gene-symbol level. To improve comparability between the RNA-seq and microarray cohorts, expression data were log2 transformed when necessary and standardized before integration.

To eliminate non-biological variations introduced by different platforms and cohorts, batch-effect correction was performed using the ComBat algorithm implemented in the sva R package. Dataset source (TCGA-STAD or GSE62254) was specified as the batch variable, and the corrected expression matrix was used for all subsequent cross-cohort analyses. The effectiveness of batch correction was evaluated by unsupervised distribution comparison and principal component clustering before and after correction.

Differential expression analyses were performed to identify genes associated with PNI and with the CXCL8/VCAM1 expression pattern. First, PNI-positive and PNI-negative samples were compared separately in the TCGA-STAD and GSE62254 cohorts. Genes showing consistent differential trends across the two public cohorts were considered PNI-related differentially expressed genes (DEGs). Second, based on the optimal expression cut-off values of CXCL8 and VCAM1, patients were stratified into a CXCL8 high + VCAM1 high group and a CXCL8 low + VCAM1 low group, followed by DEG analysis between these two groups. Differential expression analysis was performed using R-based statistical pipelines, and genes with |log2 fold change| > 1 and adjusted P < 0.05 were considered significant. Multiple testing correction was performed using the Benjamini-Hochberg method.

To prioritize biologically relevant candidates, genes associated with “perineural invasion” and “tumor–nerve interaction” were collected from Pathway Studio. These genes were imported into the STRING database to construct a protein-protein interaction (PPI) network. The network was further analyzed in Cytoscape, and hub genes were identified using the CytoHubba plugin based on network topology parameters. Candidate hub genes were then intersected with the PNI-related DEGs to define core genes for downstream analyses.

Functional enrichment analyses, including Gene Ontology (GO) and Kyoto Encyclopedia of Genes and Genomes (KEGG) pathway analyses, were performed to explore the biological processes and signaling pathways associated with the identified core gene set. In parallel, weighted gene co-expression network analysis (WGCNA) was conducted using the WGCNA R package to identify gene modules significantly associated with clinicopathological traits, including overall survival, tumor stage, and PNI status. Genes within trait-relevant modules were further examined as potential regulatory components of the PNI-associated transcriptional program.

To evaluate the relationship between the MIF–VCAM1–CXCL8 axis and the tumor microenvironment, cell-type deconvolution analysis was performed using CIBERSORTx on the TCGA-STAD cohort. Relative proportions of major stromal and immune cell populations were estimated from bulk transcriptomic profiles. Patients were stratified into high- and low-expression groups according to the optimal cut-off value of each core gene, and differences in the inferred cell fractions between groups were compared using the Wilcoxon rank-sum test.

Survival analyses were performed in the TCGA-STAD and GSE62254 cohorts independently. Kaplan-Meier curves were generated and compared using the log-rank test. Univariate and multivariable Cox proportional hazards regression models were used to estimate hazard ratios and 95% confidence intervals where appropriate. All bioinformatic analyses were conducted in R software (version 4.2.1), and major packages included sva, limma, survival, survminer, WGCNA, clusterProfiler, ggplot2, dplyr, and CIBERSORTx-related pipelines. A two-sided P < 0.05 was considered statistically significant unless otherwise specified.

The workflow is shown below:


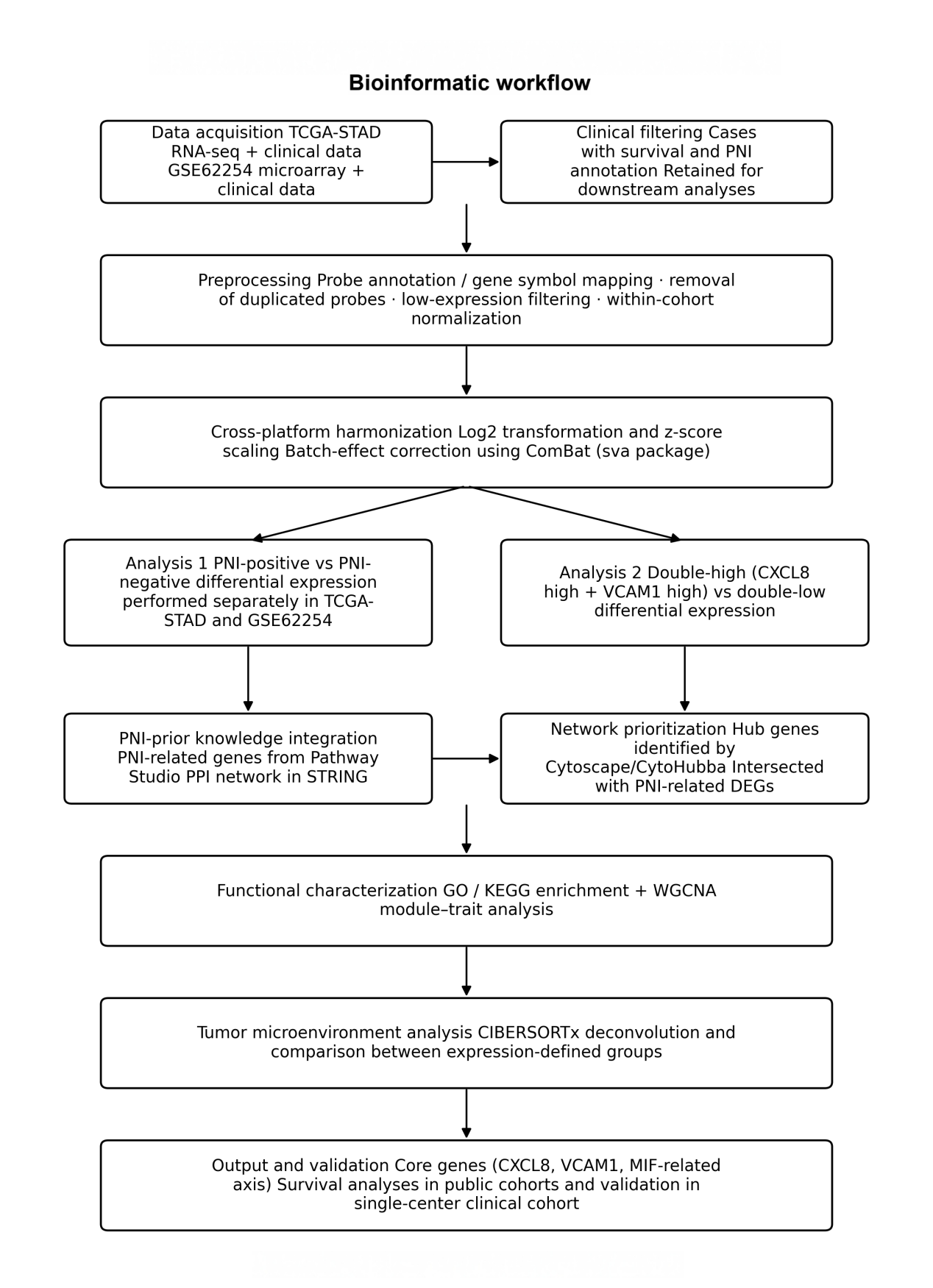

Supplement: Supplementary file 1 — Supporting File 1: advs76195‐sup‐0001‐SuppMat.docx. [file ADVS-9999-e76195-s004.docx]
