# Supplementary material for: Neuron‐Derived MIF Engages VCAM1 to Fuel a Self‐Amplifying CXCL8 Loop That Drives Perineural Invasion and Metastasis in Gastric Cancer
Source: Adv Sci (Weinh). 2026 Jun 22:e76195. Online ahead of print. doi: 10.1002/advs.76195 (PMC13337004; doi:10.1002/advs.76195)
Supplement: Supplementary file 2 — Supporting File 2: advs76195‐sup‐0002‐FigureAndTableCaption.docx. [file ADVS-9999-e76195-s003.docx]

Supplementary Figure S1: Associations between CXCL8 expression and tumor microenvironment cell composition in gastric cancer.

Box plots comparing the proportions of different tumor microenvironment (TME) cell types between patients with high and low CXCL8 expression in the TCGA-STAD cohort. CXCL8 high expression was significantly associated with increased proportions of inflammatory monocytes (****P < 0.0001) and mast cells (**P < 0.01)

, and decreased proportion of CD8^+^ T cells (*P < 0.05). No significant differences were observed for other cell populations. Statistical analysis was performed using the Wilcoxon rank-sum test. *P < 0.05, **P < 0.01, ****P < 0.0001; ns, not significant.

Supplementary Figure S2: Associations between VCAM1 expression and tumor microenvironment cell composition in gastric cancer.

Box plots comparing the proportions of different TME cell types between patients with high and low VCAM1 expression in the TCGA-STAD cohort. VCAM1 high expression was significantly associated with increased proportions of cancer-associated fibroblasts (CAFs) (****P < 0.0001), endothelial cells (****P < 0.0001), macrophages (***P < 0.001), pericytes-VSMCs (*P < 0.05), and plasma cells (****P < 0.0001). No significant differences were observed for other cell populations. Statistical analysis was performed using the Wilcoxon ranksum test. *P < 0.05, ***P < 0.001, ****P < 0.0001; ns, not significant.

Supplementary Figure S3: Associations between MIF expression and tumor microenvironment cell composition in gastric cancer.

Box plots comparing the proportions of different TME cell types between patients with high and low MIF expression in the TCGA-STAD cohort. No statistically significant differences in the proportions of the examined cell populations were observed between the two groups. Statistical analysis was performed using the Wilcoxon rank-sum test; ns, not significant.

Supplementary Figure S4: Neuronal CXCR2 blockade abrogates DRG co-culture-induced upregulation of VCAM1 and CXCL8 in gastric cancer cells.

SNU-216 gastric cancer cells were cultured alone (control) or co-cultured with rat DRG neurons in a Transwell system for 72 hours. Prior to co-culture,

DRG neurons were pretreated with 10 μM selective CXCR2 antagonist SB225002 (DRG(+)) or equal volume of DMSO vehicle control (DRG(-)) for 6

hours, followed by three thorough washes with pre-warmed PBS to completely remove unbound drug. Left: Representative Western blot images of

VCAM1 and CXCL8 protein expression in SNU-216 cells, with GAPDH as the loading control. Right: Quantitative analysis of VCAM1 and CXCL8

protein levels normalized to GAPDH. Data are presented as mean ± SD from three independent experiments. *P < 0.05, ***P < 0.001 by one-way

ANOVA with Tukey’s post hoc test.

Supplementary Figure S5: VCAM1 is required for MIF-induced activation of ERK/STAT3 signaling and upregulation of CXCL8 in gastric cancer cells.

SNU-216 gastric cancer cells were transfected with siRNA targeting VCAM1 (siVCAM1) or non-targeting control siRNA (siNC). After 48 hours, cells were treated with recombinant human MIF or PBS for 24 hours. Left: Representative Western blot images showing protein expression of VCAM1, CXCL8, total STAT3, phosphorylated STAT3 (P-STAT3), total ERK1/2, phosphorylated ERK1/2 (P-ERK1/2), with GAPDH as the loading control. Right: Quantitative analysis of protein levels. Data are presented as mean ± SD from three independent experiments. ***P < 0.001 by one-way ANOVA with Tukey’s post hoc test.

Supplementary Figure S6: Schematic representation of the results of the mice tail genotype screening gel electrophoresis experiment, (A) for Mif-flox and (B) for Nes-creERT2.

Supplementary Figure S7: Multi-level validation of knockout efficiency and Cre specificity for neuron-specific Mif conditional knockout mouse model. (A) Western blot detection of MIF protein expression in DRG tissues from Nes-cre-Miffl/fl(control) and Nes-cre+Miffl/fl(knockout mice) (n=5 per group). GAPDH was used as internal loading control; quantitative statistical analysis was shown on the right. ***P < 0.001, two-tailed Student’s t-test. (B) Quantitative real-time PCR analysis of Mif mRNA levels in DRG tissues of control and knockout mouse groups. Mif transcription was markedly depleted in knockout DRG samples. **P < 0.01, two-tailed Student’s t-test. (C) Lineage tracing assay to verify cell-type specificity of Nestin-CreERT2 recombinase in mouse gastric tissue. Confocal immunofluorescence images: tdTomato (red, marks cells with active Cre recombination), Nestin (green, pan-neuronal marker), DAPI (blue, nuclear counterstain). Merged images reveal robust co-localization between tdTomato and Nestin, confirming Cre activity is exclusively restricted to Nestin-positive neurons. Scale bars: 50 μm.

Supplementary Figure S8: A.Representative S100 immunohistochemical staining showing perineural invasion in human gastric cancer tissues (top row) and mouse gastric cancer tissues (bottom row). Scale bars: 50 μm.

Supplementary Figure S9: Validation of DRG culture purity and neuronal localization of MIF. (A) Representative immunofluorescence staining of three

independent primary DRG cultures using the neuron-specific marker Tuj1 (green). Nuclei were stained with DAPI (blue). Scale bars: 20 μm. (B) Double

immunofluorescence staining showing co-localization of MIF (green) and the neuronal marker Tuj1 (red) in DRG neurons. Nuclei were stained with

DAPI (blue). Scale bars: 20 μm.

Supplementary Table S1: Key experimental reagents and resources used in this study. Comprehensive list of all critical materials applied in the study, including qPCR primers, antibodies, chemical reagents, and animal model resources, with detailed information on supplier, catalog number, and supplementary technical specifications.

Supplementary Table S2: Univariate Cox regression analysis of CXCL8 and VCAM1 expression with overall survival in the TCGA-STAD cohort. Results of univariate Cox proportional hazards regression evaluating the prognostic association of CXCL8 and VCAM1 mRNA expression with overall survival in TCGA stomach adenocarcinoma patients, including hazard ratios (HR), 95% confidence intervals (CI), and corresponding P values.

Supplementary Table S3: Associations between CXCL8 expression levels and clinicopathological parameters in the TCGA-STAD cohort (n = 364). χ² test was used to analyze the correlations of low/high CXCL8 expression with clinical features including gender, age, T/N/M stage, pathological stage, Lauren classification, histological subtype and perineural invasion (PNI). Numbers and corresponding constituent ratios of patients in each subgroup are listed. P < 0.05 was defined as statistically significant.

Supplementary Table S4: Correlation between VCAM1 expression and clinicopathological features in the TCGA-STAD cohort (n = 364). Associations of dichotomized VCAM1 expression (low vs high) with clinical parameters were assessed using the χ² test. Patient counts and corresponding constituent percentages for each subgroup are displayed, along with χ² values and P values. P < 0.05 was considered statistically significant.

Supplementary Table S5: Correlation between MIF expression and clinicopathological characteristics in the TCGA-STAD cohort (n = 364). χ² test was applied to analyze the associations of dichotomized MIF expression (low vs high) with various clinical indexes. Patient number and constituent ratio of each subgroup, χ² values and corresponding P values are listed. Statistical significance was defined as P < 0.05.
