# Supplementary material for: Neuron‐Derived MIF Engages VCAM1 to Fuel a Self‐Amplifying CXCL8 Loop That Drives Perineural Invasion and Metastasis in Gastric Cancer
Source: Adv Sci (Weinh). 2026 Jun 22:e76195. Online ahead of print. doi: 10.1002/advs.76195 (PMC13337004; doi:10.1002/advs.76195)
Supplement: Supplementary file 3 — Supporting File 3: advs76195‐sup‐0003‐FigureS1‐S9.zip. [file ADVS-9999-e76195-s002.zip › Supplementary figure S1.pdf]

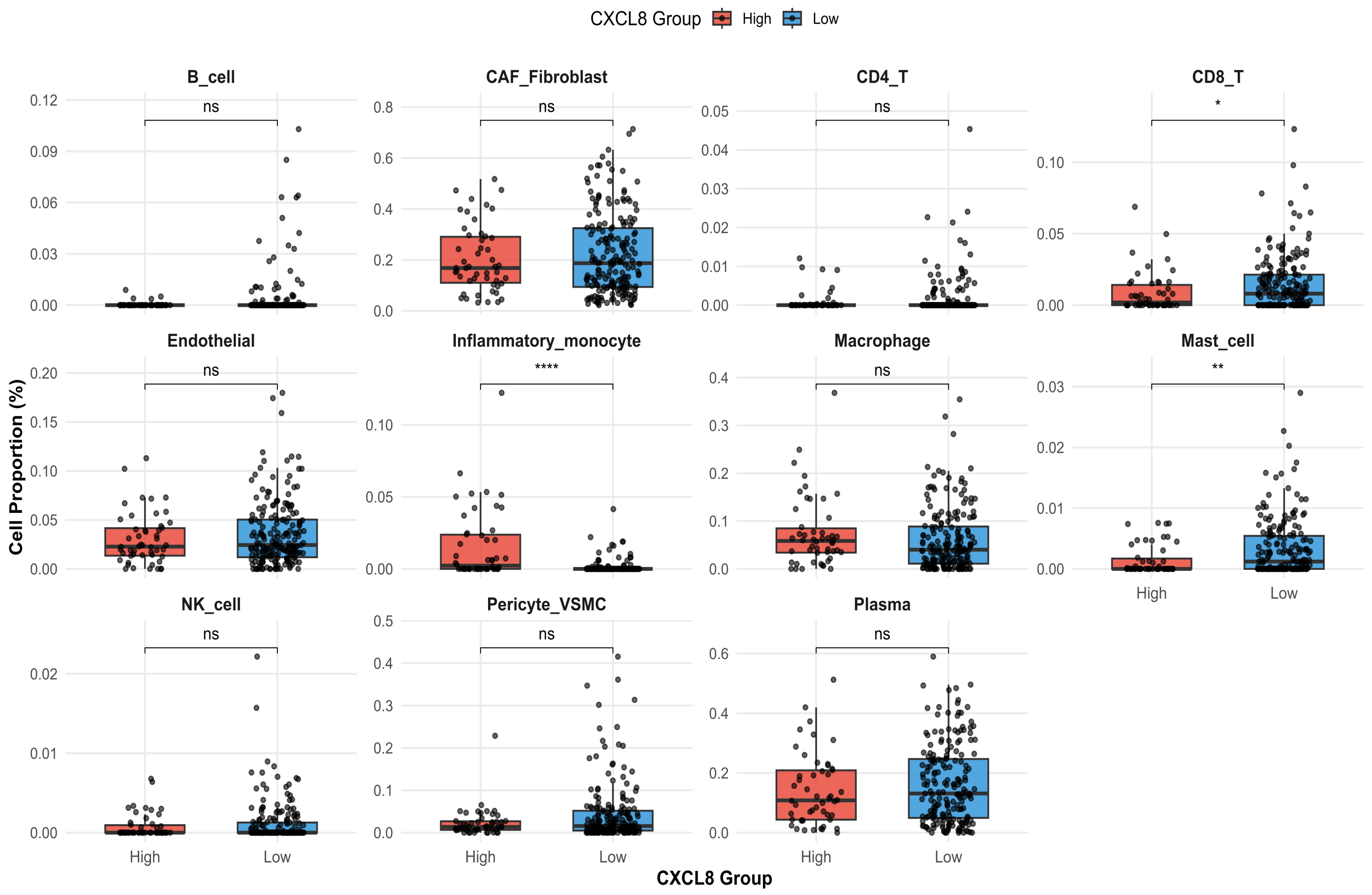

Supplementary Figure S1. Associations between CXCL8 expression and tumor microenvironment cell composition in gastric cancer

Box plots comparing the proportions of different tumor microenvironment (TME) cell types between patients with high and low CXCL8 expression in the TCGA-STAD cohort. CXCL8 high expression was significantly associated with increased proportions of inflammatory monocytes (****P < 0.0001) and mast cells (**P < 0.01), and decreased proportion of $CD8^{+}$ T cells (*P < 0.05). No significant differences were observed for other cell populations. Statistical analysis was performed using the Wilcoxon rank-sum test. *P < 0.05, **P < 0.01, ****P < 0.0001; ns, not significant.
