# Supplementary material for: Neuron‐Derived MIF Engages VCAM1 to Fuel a Self‐Amplifying CXCL8 Loop That Drives Perineural Invasion and Metastasis in Gastric Cancer
Source: Adv Sci (Weinh). 2026 Jun 22:e76195. Online ahead of print. doi: 10.1002/advs.76195 (PMC13337004; doi:10.1002/advs.76195)
Supplement: Supplementary file 3 — Supporting File 3: advs76195‐sup‐0003‐FigureS1‐S9.zip. [file ADVS-9999-e76195-s002.zip › Supplementary Figure S2.pdf]

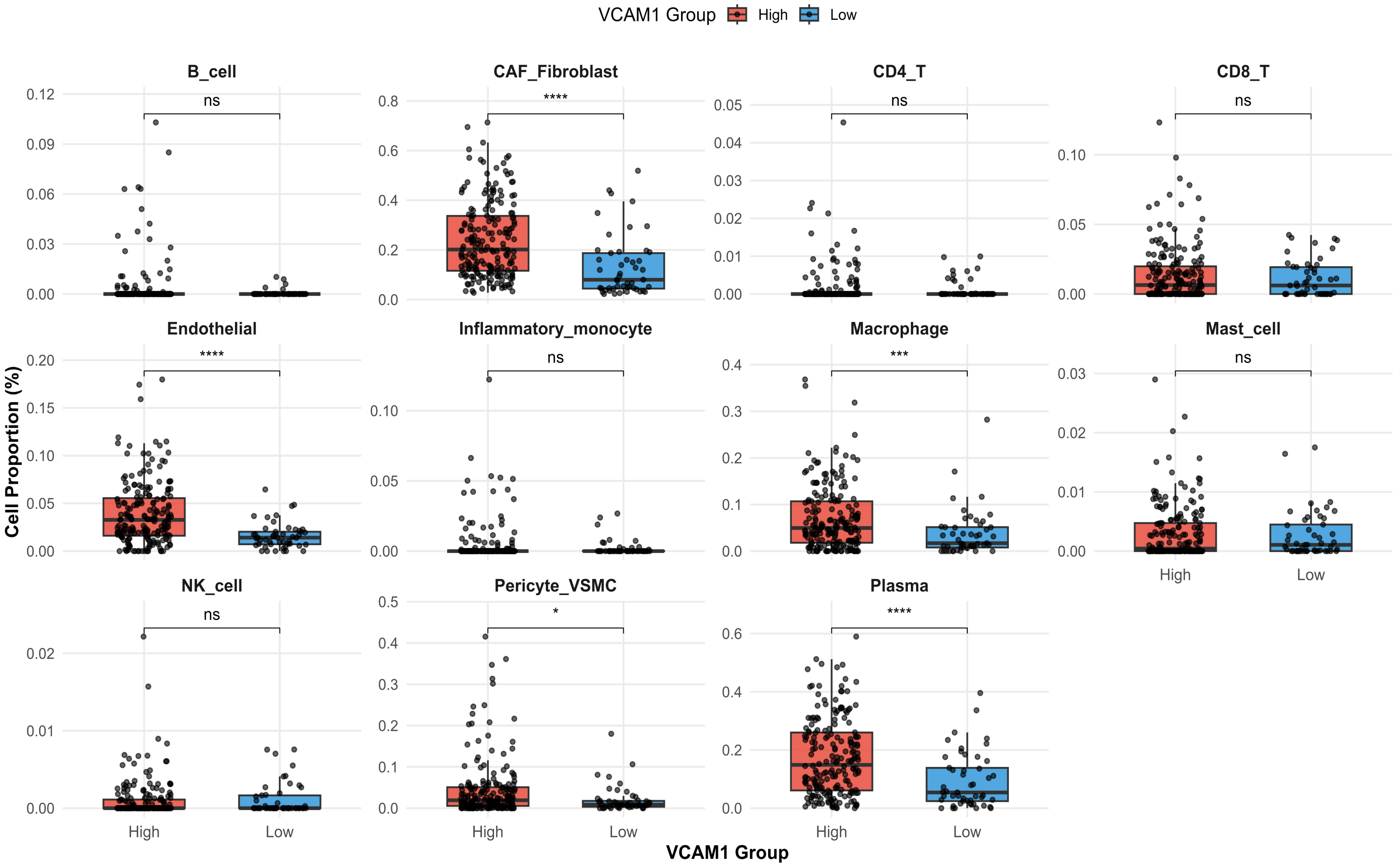

Supplementary Figure S2. Associations between VCAM1 expression and tumor microenvironment cell composition in gastric cancer

Box plots comparing the proportions of different TME cell types between patients with high and low VCAM1 expression in the TCGA-STAD cohort. VCAM1 high expression was significantly associated with increased proportions of cancer-associated fibroblasts (CAFs) (****$P < 0.0001$), endothelial cells (****$P < 0.0001$), macrophages (***$P < 0.001$), pericytes-VSMCs (*$P < 0.05$), and plasma cells (****$P < 0.0001$). No significant differences were observed for other cell populations. Statistical analysis was performed using the Wilcoxon rank-sum test. *$P < 0.05$, ***$P < 0.001$, ****$P < 0.0001$; ns, not significant.
