# Supplementary material for: Neuron‐Derived MIF Engages VCAM1 to Fuel a Self‐Amplifying CXCL8 Loop That Drives Perineural Invasion and Metastasis in Gastric Cancer
Source: Adv Sci (Weinh). 2026 Jun 22:e76195. Online ahead of print. doi: 10.1002/advs.76195 (PMC13337004; doi:10.1002/advs.76195)
Supplement: Supplementary file 3 — Supporting File 3: advs76195‐sup‐0003‐FigureS1‐S9.zip. [file ADVS-9999-e76195-s002.zip › Supplementary Figure S3.pdf]

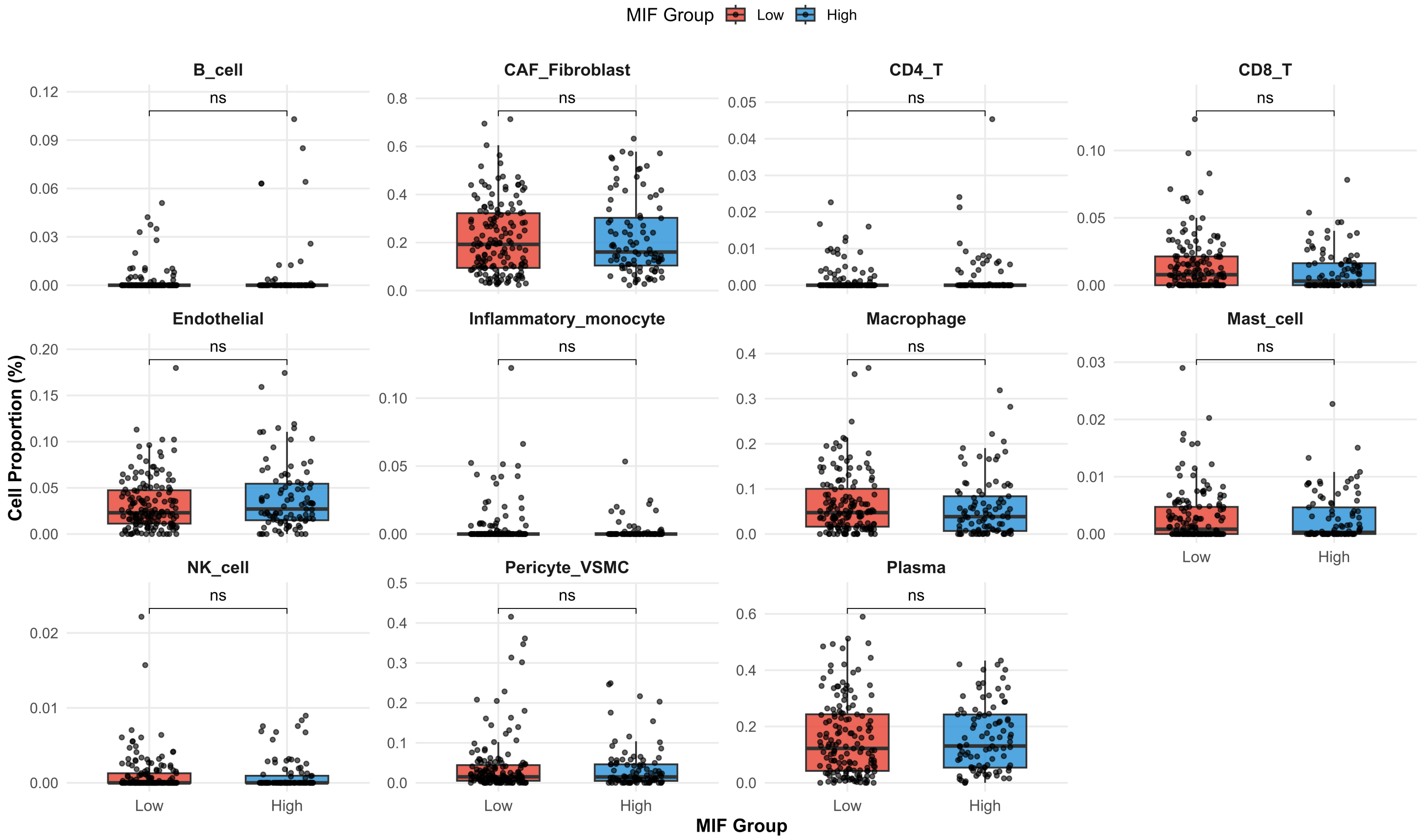

Supplementary Figure S3. Associations between MIF expression and tumor microenvironment cell composition in gastric cancer

Box plots comparing the proportions of different TME cell types between patients with high and low MIF expression in the TCGA-STAD cohort. No statistically significant differences in the proportions of the examined cell populations were observed between the two groups. Statistical analysis was performed using the Wilcoxon rank-sum test; ns, not significant.
