# Supplementary material for: Neuron‐Derived MIF Engages VCAM1 to Fuel a Self‐Amplifying CXCL8 Loop That Drives Perineural Invasion and Metastasis in Gastric Cancer
Source: Adv Sci (Weinh). 2026 Jun 22:e76195. Online ahead of print. doi: 10.1002/advs.76195 (PMC13337004; doi:10.1002/advs.76195)
Supplement: Supplementary file 3 — Supporting File 3: advs76195‐sup‐0003‐FigureS1‐S9.zip. [file ADVS-9999-e76195-s002.zip › Supplementary Figure S4.pdf]

Figure S4

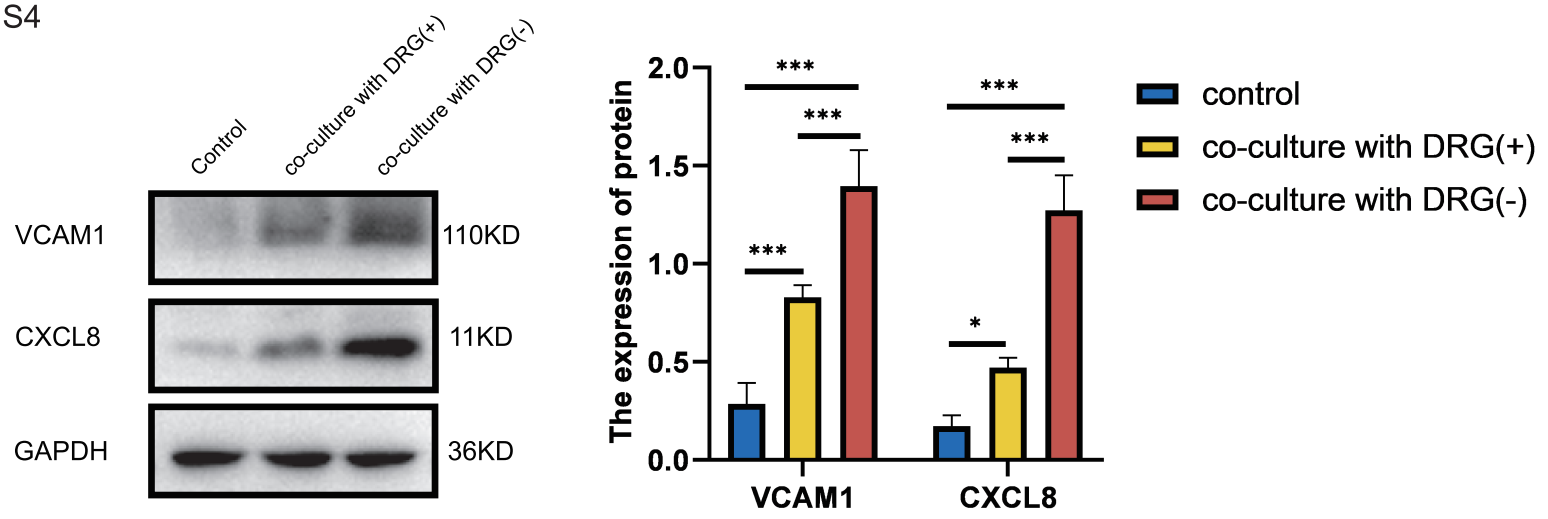

Supplementary Figure S4. Neuronal CXCR2 blockade abrogates DRG co-culture-induced upregulation of VCAM1 and CXCL8 in gastric cancer cells. SNU-216 gastric cancer cells were cultured alone (control) or co-cultured with rat DRG neurons in a Transwell system for 72 hours. Prior to co-culture, DRG neurons were pretreated with 10 μM selective CXCR2 antagonist SB225002 (DRG(+)) or equal volume of DMSO vehicle control (DRG(-)) for 6 hours, followed by three thorough washes with pre-warmed PBS to completely remove unbound drug. Left: Representative Western blot images of VCAM1 and CXCL8 protein expression in SNU-216 cells, with GAPDH as the loading control. Right: Quantitative analysis of VCAM1 and CXCL8 protein levels normalized to GAPDH. Data are presented as mean ± SD from three independent experiments. *$P < 0.05$, ***$P < 0.001$ by one-way ANOVA with Tukey’s post hoc test.
