# Supplementary material for: Neuron‐Derived MIF Engages VCAM1 to Fuel a Self‐Amplifying CXCL8 Loop That Drives Perineural Invasion and Metastasis in Gastric Cancer
Source: Adv Sci (Weinh). 2026 Jun 22:e76195. Online ahead of print. doi: 10.1002/advs.76195 (PMC13337004; doi:10.1002/advs.76195)
Supplement: Supplementary file 3 — Supporting File 3: advs76195‐sup‐0003‐FigureS1‐S9.zip. [file ADVS-9999-e76195-s002.zip › Supplementary figure S5.pdf]

Figure S5

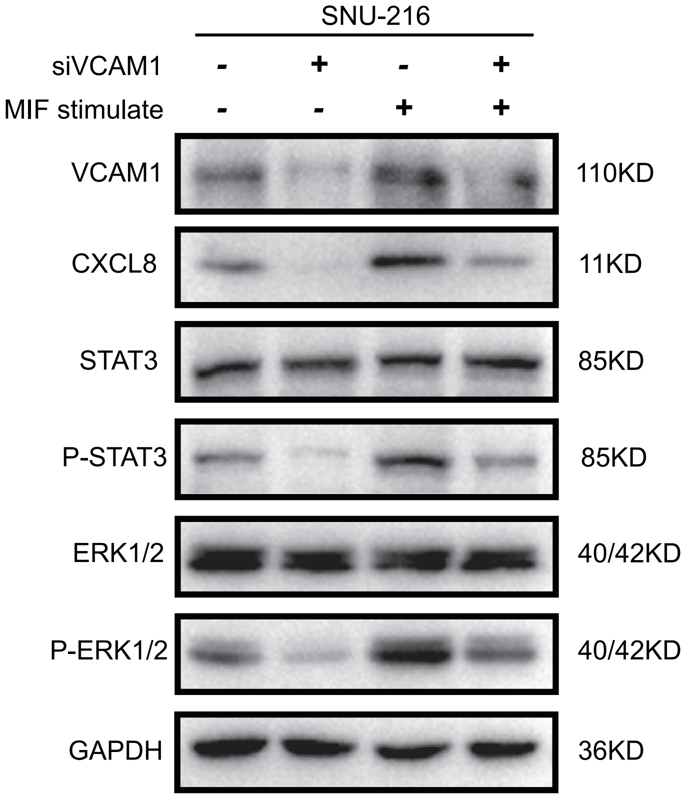

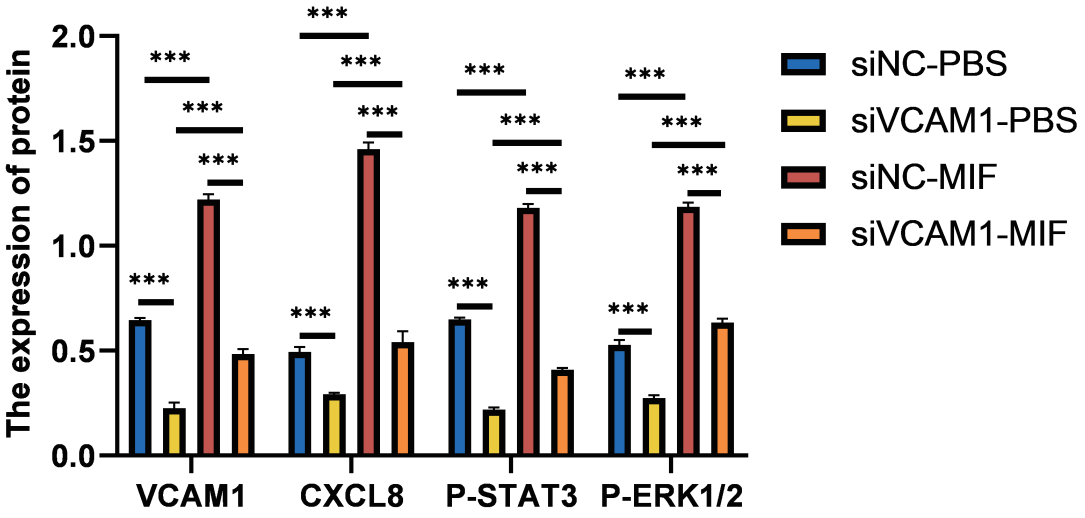

Supplementary Figure S5. VCAM1 is required for MIF-induced activation of ERK/STAT3 signaling and upregulation of CXCL8 in gastric cancer cells.

SNU-216 gastric cancer cells were transfected with siRNA targeting VCAM1 (siVCAM1) or non-targeting control siRNA (siNC). After 48 hours, cells were treated with recombinant human MIF or PBS for 24 hours. Left: Representative Western blot images showing protein expression of VCAM1, CXCL8, total STAT3, phosphorylated STAT3 (P-STAT3), total ERK1/2, phosphorylated ERK1/2 (P-ERK1/2), with GAPDH as the loading control. Right: Quantitative analysis of protein levels. Data are presented as mean ± SD from three independent experiments. ***P < 0.001 by one-way ANOVA with Tukey's post hoc test.
