# Supplementary material for: Neuron‐Derived MIF Engages VCAM1 to Fuel a Self‐Amplifying CXCL8 Loop That Drives Perineural Invasion and Metastasis in Gastric Cancer
Source: Adv Sci (Weinh). 2026 Jun 22:e76195. Online ahead of print. doi: 10.1002/advs.76195 (PMC13337004; doi:10.1002/advs.76195)
Supplement: Supplementary file 3 — Supporting File 3: advs76195‐sup‐0003‐FigureS1‐S9.zip. [file ADVS-9999-e76195-s002.zip › Supplementary Figure S7.pdf]

Figure S7

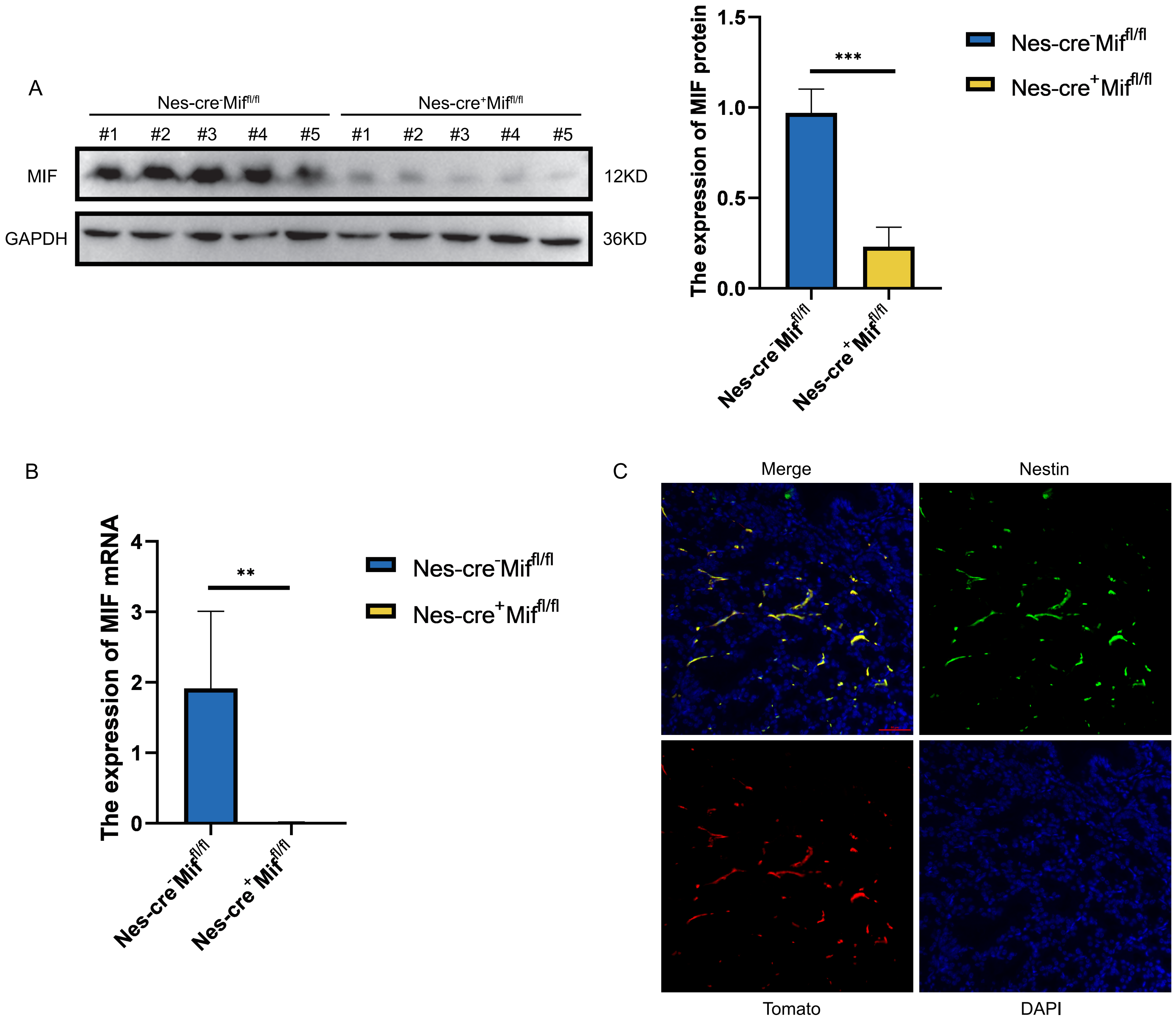

Supplementary Figure S7. Multi-level validation of knockout efficiency and Cre specificity for neuron-specific Mif conditional knockout mouse model. (A) Western blot detection of MIF protein expression in DRG tissues from Nes-cre^{-}Mif$^{fl/fl}$(control) and Nes-cre^{+}Mif$^{fl/fl}$(knockout mice) (n=5 per group). GAPDH was used as internal loading control; quantitative statistical analysis was shown on the right. ***$P < 0.001$, two-tailed Student's t-test. (B) Quantitative real-time PCR analysis of Mif mRNA levels in DRG tissues of control and knockout mouse groups. Mif transcription was markedly depleted in knockout DRG samples. **$P < 0.01$, two-tailed Student's t-test. (C) Lineage tracing assay to verify cell-type specificity of Nestin-CreERT2 recombinase in mouse gastric tissue. Confocal immunofluorescence images: tdTomato (red, marks cells with active Cre recombination), Nestin (green, pan-neuronal marker), DAPI (blue, nuclear counterstain). Merged images reveal robust co-localization between tdTomato and Nestin, confirming Cre activity is exclusively restricted to Nestin-positive neurons. Scale bars: 50 μm.
