# Supplementary material for: Neuron‐Derived MIF Engages VCAM1 to Fuel a Self‐Amplifying CXCL8 Loop That Drives Perineural Invasion and Metastasis in Gastric Cancer
Source: Adv Sci (Weinh). 2026 Jun 22:e76195. Online ahead of print. doi: 10.1002/advs.76195 (PMC13337004; doi:10.1002/advs.76195)
Supplement: Supplementary file 3 — Supporting File 3: advs76195‐sup‐0003‐FigureS1‐S9.zip. [file ADVS-9999-e76195-s002.zip › Supplementary Figure S8.pdf]

Figure S8

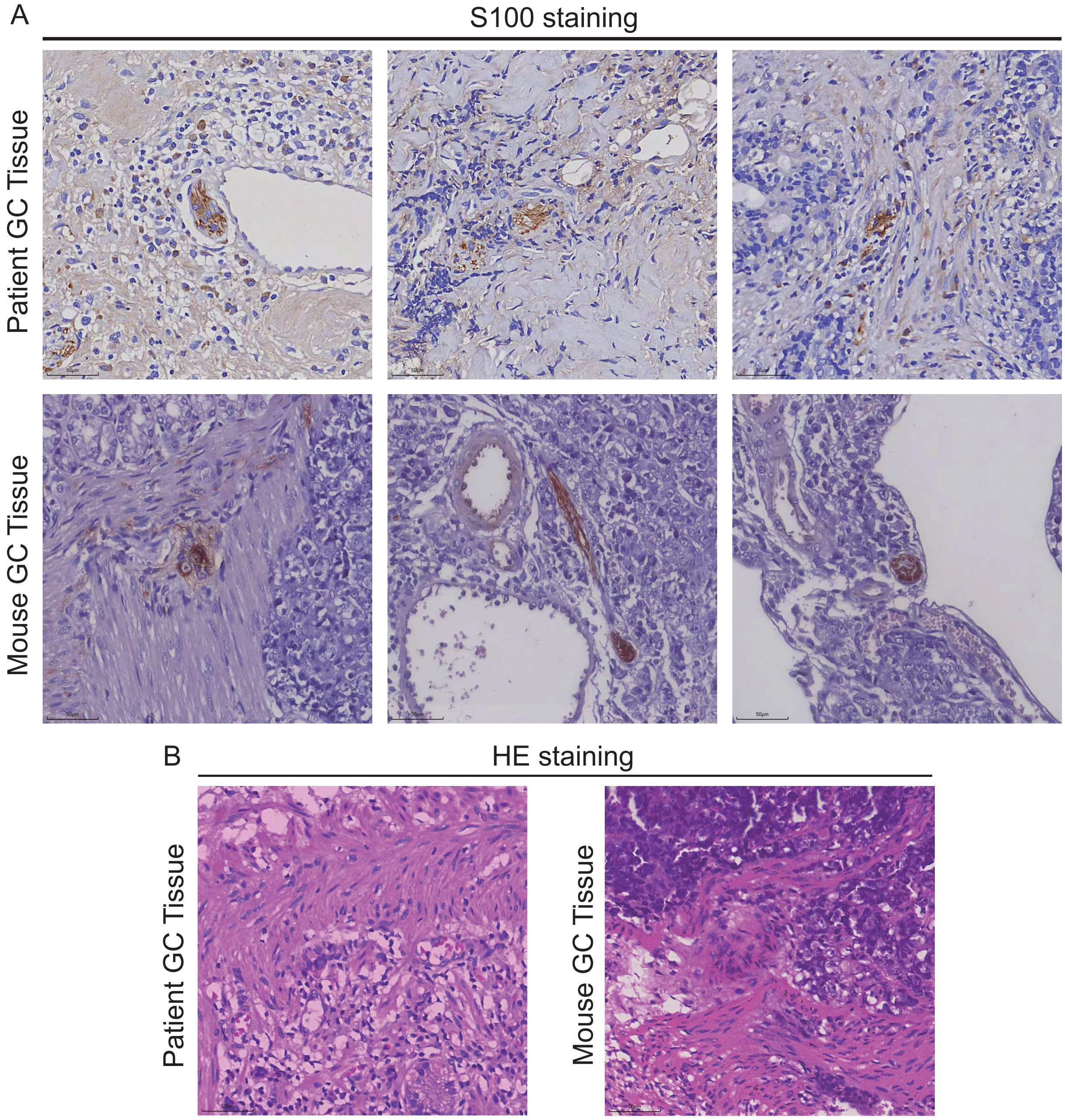

Figure S8: A.Representative S100 immunohistochemical staining showing perineural invasion in human gastric cancer tissues (top row) and mouse tumor tissues (bottom row). Scale bars: 50 μm. B.Representative HE staining of patient GC tissue(left) and mouse GC tissue(right). Scale bars: 50 μm.
