# Supplementary material for: Neuron‐Derived MIF Engages VCAM1 to Fuel a Self‐Amplifying CXCL8 Loop That Drives Perineural Invasion and Metastasis in Gastric Cancer
Source: Adv Sci (Weinh). 2026 Jun 22:e76195. Online ahead of print. doi: 10.1002/advs.76195 (PMC13337004; doi:10.1002/advs.76195)
Supplement: Supplementary file 3 — Supporting File 3: advs76195‐sup‐0003‐FigureS1‐S9.zip. [file ADVS-9999-e76195-s002.zip › Supplementary figure S9.pdf]

Figure S9

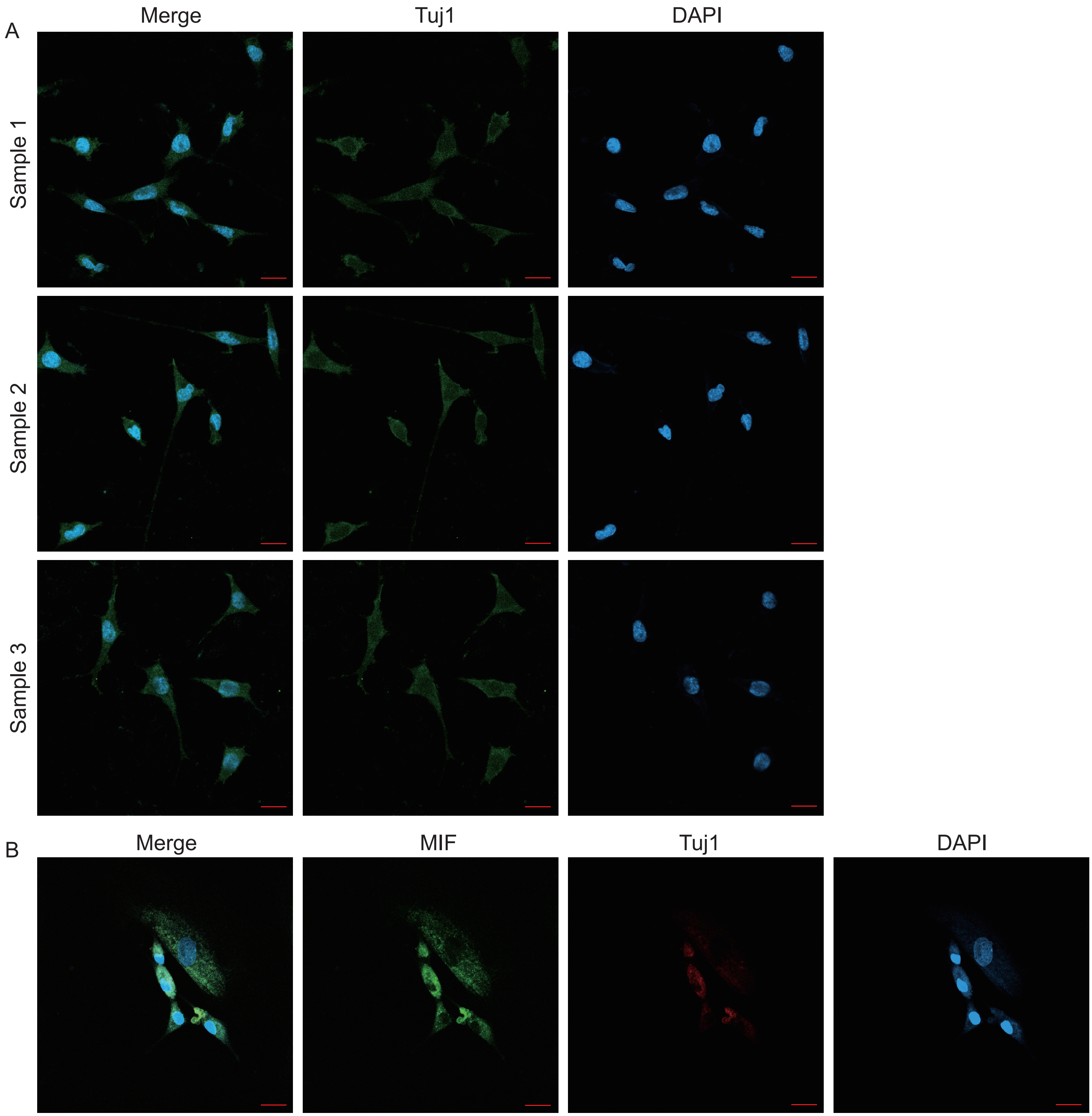

Supplementary Figure S9. Validation of DRG culture purity and neuronal localization of MIF. (A) Representative immunofluorescence staining of three independent primary DRG cultures using the neuron-specific marker Tuj1 (green). Nuclei were stained with DAPI (blue). Scale bars: 20 μm. (B) Double immunofluorescence staining showing co-localization of MIF (green) and the neuronal marker Tuj1 (red) in DRG neurons. Nuclei were stained with DAPI (blue). Scale bars: 20 μm.
