# Supplementary material for: Neuron‐Derived MIF Engages VCAM1 to Fuel a Self‐Amplifying CXCL8 Loop That Drives Perineural Invasion and Metastasis in Gastric Cancer
Source: Adv Sci (Weinh). 2026 Jun 22:e76195. Online ahead of print. doi: 10.1002/advs.76195 (PMC13337004; doi:10.1002/advs.76195)
Supplement: Supplementary file 4 — Supporting File 4: advs76195‐sup‐0004‐TableS1‐S5.zip. [file ADVS-9999-e76195-s001.zip › Supplementary Table S3.pdf]

Supplementary Table S3

**Characteristics**	***n***	**CXCL8 expression (*n* =364)**		X^2	***P***
		**Low**	**High**		
Gender				6.33	0.0118
Female	106	48 (45.3%)	58 (54.7%)		
Male	258	81 (31.4%)	177 (68.6%)		
Age(year)				1	0.3171
<60	115	45 (39.1%)	70 (60.9%)		
≥60	249	84 (33.7%)	165 (66.3%)		
T Stage				0.02	0.8908
T1-T2	176	63 (35.8%)	113 (64.2%)		
T3-T4	188	66 (35.1%)	122 (64.9%)		
N Stage				1.89	0.1696
N0-N1	201	65 (32.3%)	136 (67.7%)		
N2-N3	163	64 (39.3%)	99 (60.7%)		
M Stage				1.56	0.2114
M0	329	114 (34.7%)	215 (65.3%)		
M1	28	13 (46.4%)	15 (53.6%)		
pStage				0.48	0.4896
I-II	147	48 (32.7%)	99 (67.3%)		
III-IV	210	76 (36.2%)	134 (63.8%)		
Lauren type				5.53	0.0629
Diffuse	137	58 (42.3%)	79 (57.7%)		
Intestinal	199	61 (30.7%)	138 (69.3%)		
Mix	9	2 (22.2%)	7 (77.8%)		
Pathohistological type				0.42	0.5149
Adenocarcinoma	316	114 (36.1%)	202 (63.9%)		
Other	48	15 (31.2%)	33 (68.8%)		
Perineural Invasion				5.44	0.0197
Absent	213	65 (30.5%)	148 (69.5%)		
Present	151	64 (42.4%)	87 (57.6%)		
