# Supplementary material for: Neuron‐Derived MIF Engages VCAM1 to Fuel a Self‐Amplifying CXCL8 Loop That Drives Perineural Invasion and Metastasis in Gastric Cancer
Source: Adv Sci (Weinh). 2026 Jun 22:e76195. Online ahead of print. doi: 10.1002/advs.76195 (PMC13337004; doi:10.1002/advs.76195)
Supplement: Supplementary file 4 — Supporting File 4: advs76195‐sup‐0004‐TableS1‐S5.zip. [file ADVS-9999-e76195-s001.zip › Supplementary Table S4.pdf]

Supplementary Table S4

**Characteristics**	***n***	**VCAM1 expression (*n* =364)**		X^2	***P***
		**Low**	**High**		
Gender				1.14	0.2857
Female	106	51 (48.1%)	55 (51.9%)		
Male	258	140 (54.3%)	118 (45.7%)		
Age(year)				3.55	0.0596
<60	115	52 (45.2%)	63 (54.8%)		
≥60	249	139 (55.8%)	110 (44.2%)		
T Stage				13.74	0.0002
T1-T2	176	110 (62.5%)	66 (37.5%)		
T3-T4	188	81 (43.1%)	107 (56.9%)		
N Stage				4.05	0.0443
N0-N1	201	115 (57.2%)	86 (42.8%)		
N2-N3	163	76 (46.6%)	87 (53.4%)		
M Stage				7.07	0.0078
M0	329	180 (54.7%)	149 (45.3%)		
M1	28	8 (28.6%)	20 (71.4%)		
pStage				8.57	0.0034
I-II	147	91 (61.9%)	56 (38.1%)		
III-IV	210	97 (46.2%)	113 (53.8%)		
Lauren type				45.43	<0.0001
Diffuse	137	41 (29.9%)	96 (70.1%)		
Intestinal	199	133 (66.8%)	66 (33.2%)		
Mix	9	3 (33.3%)	6 (66.7%)		
Pathohistological type				19.37	<0.0001
Adenocarcinoma	316	180 (57%)	136 (43%)		
Other	48	11 (22.9%)	37 (77.1%)		
Perineural Invasion				5.73	0.0167
Absent	213	123 (57.7%)	90 (42.3%)		
Present	151	68 (45%)	83 (55%)		
