# Supplementary material for: Neuron‐Derived MIF Engages VCAM1 to Fuel a Self‐Amplifying CXCL8 Loop That Drives Perineural Invasion and Metastasis in Gastric Cancer
Source: Adv Sci (Weinh). 2026 Jun 22:e76195. Online ahead of print. doi: 10.1002/advs.76195 (PMC13337004; doi:10.1002/advs.76195)
Supplement: Supplementary file 4 — Supporting File 4: advs76195‐sup‐0004‐TableS1‐S5.zip. [file ADVS-9999-e76195-s001.zip › Supplementary Table S5.pdf]

Supplementary Table S5

**Characteristics**	***n***	**MIF expression (*n* =364)**		X^2	***P***
		**Low**	**High**		
Gender				0.32	0.5708
Female	106	54 (50.9%)	52 (49.1%)		
Male	258	123 (47.7%)	135 (52.3%)		
Age(year)				3.32	0.0684
<60	115	64 (55.7%)	51 (44.3%)		
≥60	249	113 (45.4%)	136 (54.6%)		
T Stage				4.93	0.0264
T1-T2	176	75 (42.6%)	101 (57.4%)		
T3-T4	188	102 (54.3%)	86 (45.7%)		
N Stage				1	0.3176
N0-N1	201	93 (46.3%)	108 (53.7%)		
N2-N3	163	84 (51.5%)	79 (48.5%)		
M Stage				0.92	0.3382
M0	329	157 (47.7%)	172 (52.3%)		
M1	28	16 (57.1%)	12 (42.9%)		
pStage				3.46	0.0628
I-II	147	63 (42.9%)	84 (57.1%)		
III-IV	210	111 (52.9%)	99 (47.1%)		
Lauren type				9.14	0.0104
Diffuse	137	77 (56.2%)	60 (43.8%)		
Intestinal	199	90 (45.2%)	109 (54.8%)		
Mix	9	1 (11.1%)	8 (88.9%)		
Pathohistological type				4.26	0.039
Adenocarcinoma	316	147 (46.5%)	169 (53.5%)		
Other	48	30 (62.5%)	18 (37.5%)		
Perineural Invasion				4.15	0.0416
Absent	213	94 (44.1%)	119 (55.9%)		
Present	151	83 (55%)	68 (45%)		
